# ATP hydrolysis and nucleotide exit enhance maltose translocation in the MalFGK_2_E importer

**DOI:** 10.1038/s41598-021-89556-y

**Published:** 2021-05-19

**Authors:** Bárbara Abreu, Carlos Cruz, A. Sofia F. Oliveira, Cláudio M. Soares

**Affiliations:** 1 ITQB NOVA, Instituto de Tecnologia Química E Biológica António Xavier, Universidade Nova de Lisboa, Oeiras, Portugal; 2 School of Biochemistry and Centre for Computational Chemistry, University of Bristol, Bristol, UK

**Keywords:** Biophysics, Computational biophysics, Structural biology, Molecular modelling, Biochemistry, Proteins, Membrane proteins

## Abstract

ATP binding cassette (ABC) transporters employ ATP hydrolysis to harness substrate translocation across membranes. The *Escherichia coli* MalFGK_2_E maltose importer is an example of a type I ABC importer and a model system for this class of ABC transporters. The MalFGK_2_E importer is responsible for the intake of malto-oligossacharides in *E.coli.* Despite being extensively studied, little is known about the effect of ATP hydrolysis and nucleotide exit on substrate transport. In this work, we studied this phenomenon using extensive molecular dynamics simulations (MD) along with potential of mean force calculations of maltose transport across the pore, in the pre-hydrolysis, post-hydrolysis and nucleotide-free states. We concluded that ATP hydrolysis and nucleotide exit trigger conformational changes that result in the decrease of energetic barriers to maltose translocation towards the cytoplasm, with a concomitant increase of the energy barrier in the periplasmic side of the pore, contributing for the irreversibility of the process. We also identified key residues that aid in positioning and orientation of maltose, as well as a novel binding pocket for maltose in MalG. Additionally, ATP hydrolysis leads to conformations similar to the nucleotide-free state. This study shows the contribution of ATP hydrolysis and nucleotide exit in the transport cycle, shedding light on ABC type I importer mechanisms.

## Introduction

ATP binding cassette (ABC) proteins are one of the largest protein superfamilies, being ubiquitous in all domains of life. In common they all share two domains—the nucleotide binding domains (NBDs), that possess characteristic sequences that play a role in ATP binding and hydrolysis^1^, allowing ABC proteins to fulfil their function. ABC transporters are a subclass of ABC proteins responsible for the translocation of various substrates across membranes, making use of ATP hydrolysis to harness transport. In addition to the NBDs, ABC transporters also contain transmembrane domains (TMDs) that bind and translocate the substrates^2,3^. In this way, these proteins can either act as importers or exporters, depending on directionality of the transport. ABC transporters play key roles in various processes such as drug excretion in bacteria and cancer cells, lipid export for membrane building and assembling, nutrient intake and even maintenance of transmembrane gradients^4^.

**Figure 1:**
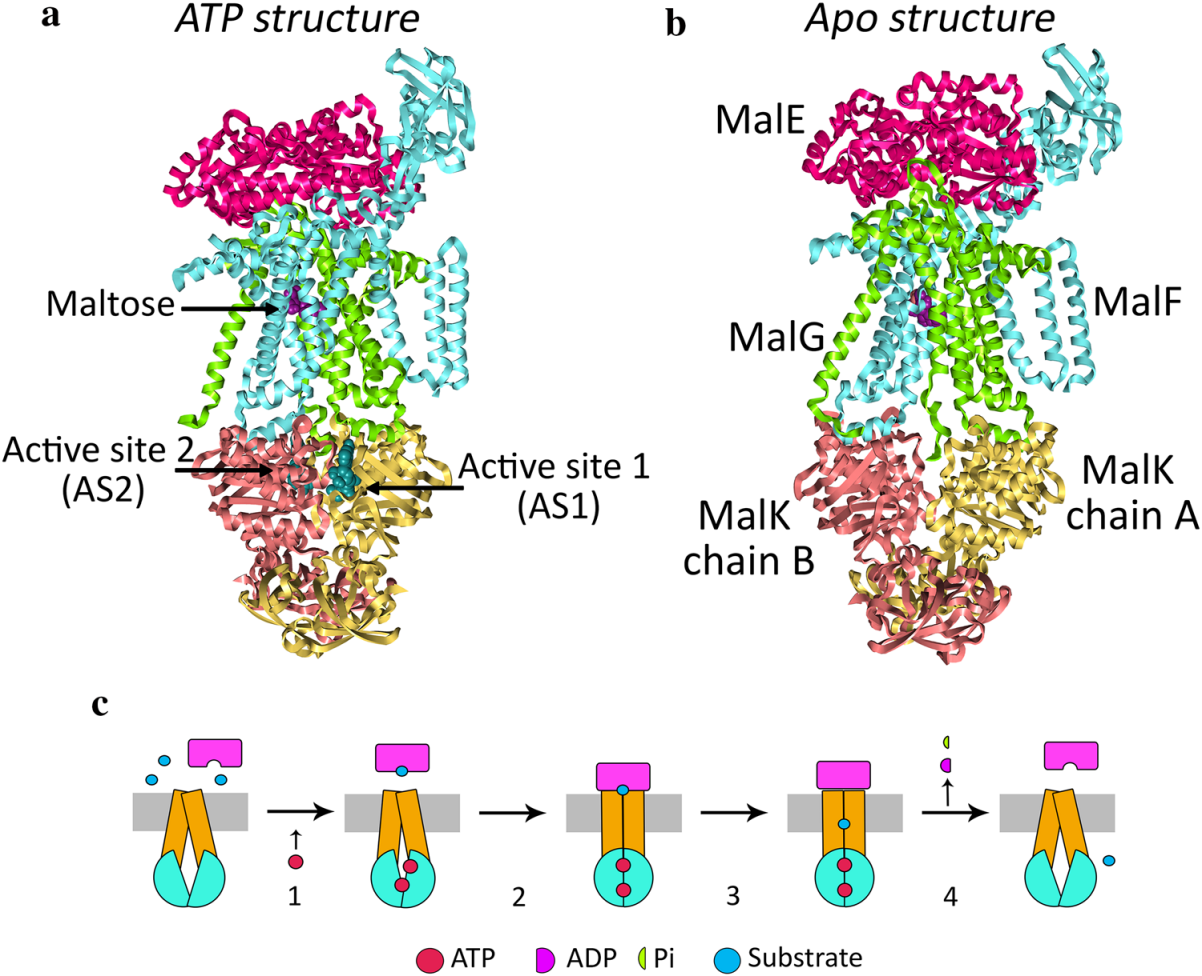
Structures used in this work. (**a**) Initial structure for the simulating the ATP state. This structure contains an ATP analogue bound in the NBDs. The post-hydrolysis state was generated from this structure. PBD code: 3RLF. (**b**) Initial structure used to mimic the protein after nucleotide exit. This structure reflects the pre-translocation state and can be used to extrapolate about the state after nucleotide exit. PBD code: 3PV0. The MalE is represented in bright pink, MalF in cyan, MalG in green and MalK in yellow and salmon. ATP is represented in teal spheres and maltose in purple spheres. (**c**) Transport cycle in the maltose importer. MalE is represented in purple, the TMDs in orange, the NBDs in cyan, ATP in red spheres, maltose as a blue sphere and ADP and Pi as purple and green semi-circles. 1- ATP binding and MalE loading with maltose. 2- MalE binding to the complex. 3- Maltose diffusion in the complex. 4- ATP hydrolysis, release of the substrate and hydrolysis products and transporter reset.

ABC importers responsible for intake of molecules, such as nutrients and metals are exclusive of bacteria^2^. In addition to the NBDs and TMDs, they also have substrate binding proteins (SBPs) that scavenge the substrate from the extracellular medium and deliver it to the transmembrane complex. Most importers are associated with the intake of nutrients and some are even involved in pathogenicity, such as the zinc importer ZnuABC present in *Brucella abortus*^5,6^. They can be divided in three subclasses: the type I, type II and type III importers, which differ in their structural features^3^). The *Escherichia coli* MalFGK_2_E importer is a type I importer located in the inner membrane responsible for the transport of maltose and malto-oligossacharides from the periplasm. It is a heterodimer, constituted by five subunits: two copies of MalK, which constitute the NBD dimer, MalF and MalG are part of the TMDs and the substrate-binding protein – MalE^7^. Figure 1 shows the structure of MalFGK_2_E in the two distinct conformations used in this work. This importer is a model system for type I ABC importers, being extensively studied over the years. Substrate translocation in ABC transporters has been explained on basis on the alternating-access model, in which the transporter alternates between an outward-facing conformation and an inward-facing one^8^. Considering this model, in the outward-facing state, the TMDs are oriented in such a way that the transmembrane cavity is exposed towards the periplasm (Fig. 1a), while the inward-facing state contains the transmembrane cavity exposed to the cytoplasm (Fig. 1b). MalE delivers the substrate to the transmembrane complex in the outward-facing form.

The transport cycle of maltose transport starts with the delivery of maltose to the complex, mediated by MalE (Fig. 1c–step 1). This first step is disputed because there are two different models to explain substrate delivery: the first one defends that there is binding of loaded MalE to the complex leading to the outward-facing conformation^7^. The second model proposes that unliganded MalE binds to the complex, and along with ATP binding, generates the outward-facing conformation^9^. Afterwards, maltose binds to the MalE-MalFGK2 complex, via MalE (Fig. 1c -steps 2 and 3)^9^. Following maltose loading to the complex, ATP hydrolysis triggers its opening causing the conversion to the inward facing form and maltose release. In this paper we will study the events happening on this latter stage (Fig. 1c- step 4).

Numerous experimental studies, along with simulation studies, have managed to characterize many of the molecular details of transport process, but the sequence of events that result from ATP hydrolysis and their corresponding molecular details, remains somewhat elusive. Nonetheless, EPR and cryo-EM data show that ATP hydrolysis results in a structure with a semi-open NBD dimer^10–12^. Moreover, kinetic studies proposed that release of phosphate is the rate limiting step and that maltose accelerates release of phosphate and ATP turnover^13^. Previous MD studies on the maltose MalFGK_2_E have also versed several stages of the transporter function. For instance, it was discovered that MalE binding decreases the energetic barrier for MalFGK_2_E complex closure, promoting the movement of the MalF and MalG helices towards the center^14^. In this way, the presence of MalE stabilizes the pre-translocation state. On the other hand, in the absence of MalE, the transmembrane helices of MalF arrange in a way that blocks the translocation pathway. MalE binding also suppresses fluctuation of the P2-loop of MalF^15^. The effect of nucleotide binding to the NBDs was also simulated, as well as its energetic and mechanistic charcterization^15–18^. ATP binding in both pockets induces the closed form of the NBD dimer, while ATP hydrolysis triggers dimer opening^16^. Other simulation studies versed the communication between NBDs and TMDs^19,20^ and concluded that the coupling helices and the Q-loops are essential for transmitting the impact of ATP hydrolysis from the NBDs to the transmembrane domains. Additionally, the mechanism of ATP hydrolysis was also investigated^21,22^. The authors suggested that ATP hydrolysis proceeds via a dissociative mechanism with a trigonal bipyramidal transition state. The conformational changes triggered by ATP hydrolysis in the complex were also studied, confirming the role of the coupling helices in transmitting the energy created by ATP hydrolysis^23^. So far, these studies characterized events that happen until ATP hydrolysis (included) and focused on the transformations suffered by the protein.

The present study aims to shed light on the process of substrate translocation. We investigate the consequences of ATP hydrolysis and nucleotide exit on the substrate transport, and on the molecular interactions between maltose and the protein complex, from the structural and energetic viewpoints. Regarding the full transport cycle, as portrayed in Fig. 1c, our study verses events on the last step of the process, when ATP is hydrolysed, and the substrate is released. To this end, we will use extensive molecular dynamics simulations of the maltose importer with maltose bound in the transmembrane domains with ATP, ADP.Pi and in the absence of nucleotides, henceforth designated as the Apo state, this latter one intended to mimic the state after hydrolysis and after nucleotide exit. The ATP and ADP.Pi states will be generated from the structure with an ATP analogue bound (Fig. 1a), while the Apo state will be created from the pre-translocation structure without nucleotides (Fig. 1b).

Even though X-ray structures containing the bound maltose (Fig. 1a,b) and even longer malto-oligassacharides^24^ provide details about substrate binding, MD simulations allow a dynamic characterization of the binding and transport processes, along with the study of impact of ATP hydrolysis on the latter.

The main findings of our study are that ATP hydrolysis significantly lowers the energetic barriers for substrate translocation, and that nucleotide departure has a very similar effect as hydrolysis. Additionally, we have identified the protein conformational changes that are responsible for this effect. The most relevant conformational changes happen on key transmembrane helices, that result in increased substrate diffusion towards the intracellular medium. Potential of mean force (PMF) calculations confirmed that hydrolysis decreases the energetic barriers for the transport process and enhances its irreversibility.

## Results and discussion

### Structural stability of MalFGK_2_E

In this work we simulated the *E. coli* MalFGK_2_E importer in different states of the transport cycle: the pre-hydrolysis state with ATP bound to the NBDs, the post-hydrolysis state with ADP and phosphate bound and an Apo (nucleotide-free) state.

In order to evaluate the structural stability of these simulations, the temporal evolution of the C-α RMSD and the percentage of retained secondary structure were monitored, along with a visual inspection of the trajectories. Regarding the ATP state, the C-α RMSD of the full complex reached a value of 0.35 nm at 40 ns, and a small drift was observed that increased the RMSD values up to 0.4 nm (Fig. S1). When observing the behaviour for each subunit it is possible to observe that the C-α RMSD of MalE and MalG stabilized around 0.2 nm (Figs. S3 and S4). The two MalK chains show higher values, around 0.25 nm and a stable evolution (Figs. S5 and S6). In contrast, the C-α RMSD of MalF shows a similar behaviour to the C-α of the full complex, reaching values around 0.3 nm and a similar drift behaviour. After a visual inspection of the trajectories, we observed that the P2 loop of MalF displays rigid-body motions, varying its position around MalE (Fig. S7). Another evidence that the complex has retained its structure throughout the simulations, is the evolution the percentage of retained native secondary structure, in comparison with the initial structure (Fig. S2), that remained stable around 95% from 40 ns onwards. Therefore, we have considered the first 40 ns of simulation as an equilibration period and were discarded. When looking at the post-hydrolysis state, a similar behaviour is observed, with the whole complex reaching C-α RMSD values around 0.35 nm, but without any significant drift (Fig. S1). MalE and MalG show again the lowest values, around 0.2 nm (Figs. S3 and S4), with a stable evolution. However, in this state, both MalK chains show higher C-α RMSD values (around 0.3 nm) than the MalF subunit (around 0.28 nm), and closer to the whole complex (Figs. S5 to S7). A visualization of the trajectories showed that significant conformational changes happen to MalK, such as the active site (AS) opening, rigid body motions back and forth of regulatory domains in the *xy* plane and increased flexibility of the C-terminal segments. This resulted in a significantly higher RMSD in comparison with its ATP counterpart. During the production MD, the transmembrane domains have adopted a more relaxed conformation, that led to a pore radius decrease in the periplasmic side and increase at the cytoplasmic side (Fig. S8), allowing the study of maltose translocation. In a similar way to the pre-hydrolysis state, the first 100 ns of simulation were discarded. This state also retained approximately 95% of its secondary structure (Fig. S2). The nucleotide-free Apo state started from an inward-facing structure. Nonetheless, within the first 20 ns of simulation, a spontaneous approximation of the TMDs was observed leading to a structure more similar to the outward-facing state. A similar behaviour was reported by Weng et al.^14^. This resulted in an increased time of equilibration, in which the first 150 ns were discarded. This closure was also reflected in pore radius, in which there was a radius increase of the periplasmic side, with a concomitant decrease on the cytoplasmic side (Fig. S8). The full complex C-α RMSD oscillates around 0.48 nm, stabilizing from 150 ns onwards (Fig. S1). On the other hand, MalE shows the lowest C-α RMSD, around 0.19 nm, while the remaining subunits show values centred in 0.3 nm, with some oscillations. The percentage of retained secondary structure is at least 96% (Fig. S2).

### Structural effects of hydrolysis and nucleotide exit

#### On nucleotide binding domains

The nucleotide binding domains of every ABC transporter play a key role in controlling the protein function, being responsible for ATP binding and hydrolysis. The NBD dimer behaviour changes upon the presence or absence of nucleotide. Previous experimental data show that ATP binding to the NBD dimer induces the closed form^7,9,13,25^. Our simulations confirm these findings because the pre-hydrolysis state has always shown a closed NBD dimer. On the other hand, the presence of ADP increases the NBDs separation, and the complete absence of nucleotide leads to a further increase^7,12,25^. These motions on the NBDs are reflected throughout the entire complex and drive conformational changes that lead to substrate movement. Therefore, by studying the degree of separation between the NBD monomers, it is possible to assess the evolution of the conformational behaviour of the complex. We defined the active site 1 (AS1) as the catalytic pocket that contains the MalK chain B Walker A motif and the chain A ABC motif. The chain B is coupled to MalF, while chain A is coupled to MalG (Fig. 1a). The active site 2 (AS2) is composed by the chain A Walker A motif and by the chain B ABC motif (Fig. 1b).

**Figure 2:**
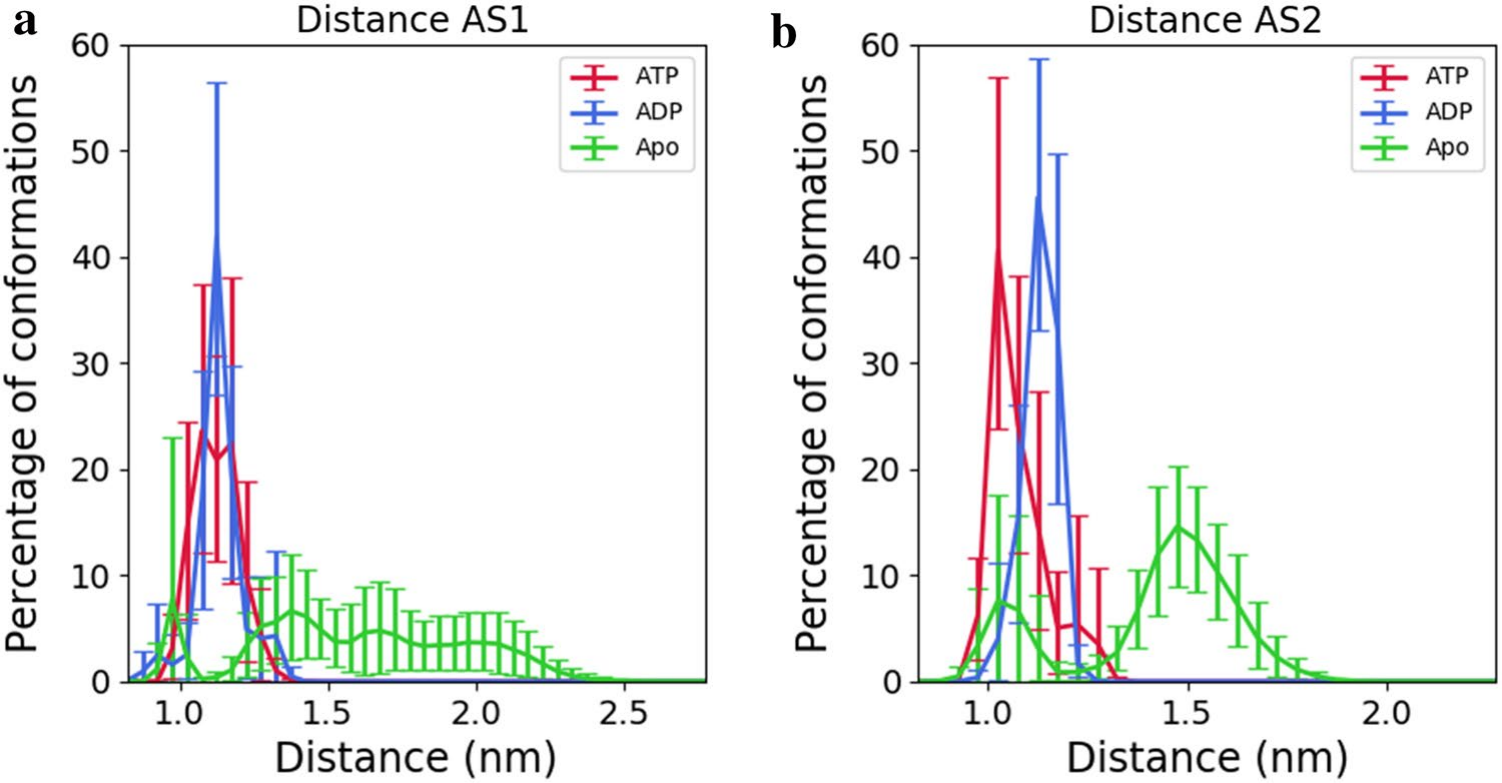
Distance of active site opening in both ATP binding pockets. AS1- Active site 1, AS2-Active site 2. This distance is measured between the C-α atoms of Walker A and the ABC motifs of the active site. The ATP state is represented in red, the ADP in blue and the Apo state without nucleotide in green. The last 260 ns of each trajectory were used for analysis. The error bars correspond to the 95% confidence interval obtained by bootstrapping. The AS1 is formed by the MalK chain (**b**) Walker (**a**) motif and the chain (**a**) ABC motif, while the AS2 is formed by the chain (**a**) Walker (**a**) motif and by the chain (**b**) ABC motif.

Figure 2 shows the distance distributions between the Walker A and the ABC motifs (using C-α atoms only) in each pocket, in all states. This measure reflects the opening degree of the ATP binding pocket, allowing to assess the evolution of the conformational changes. Is possible to observe that both pockets show distinct behaviours, regardless of the state. For the active site 1 (AS1), the distributions of ATP and ADP states overlap, while the Apo state shows a much more disperse distribution ranging from 0.8 to 2.6 nm. Regarding the active site 2 (AS2) pocket, an opening increase was observed after hydrolysis as seen by the ATP and ADP distributions. In the Apo state the total distribution is clustered in two peaks, one of them overlapping with the ATP state in the region of 0.8 to 1.2 nm, while the other spans the distances between 1.2 and 1.8 nm. Despite the Apo distribution showing wider distributions, it is important to recall that this state started in a wide open pocket conformation and its time evolution showed that it has tendency to close.

The asymmetry in pocket behaviour can be explained by the different nature of the transmembrane domains MalF and MalG, which interact with the NBDs via the coupling helices. This behaviour is enhanced in our simulations upon hydrolysis. Asymmetry in the behaviour of the nucleotide binding pockets, was also observed in cryo-EM experiments, in the presence of ADP-phosphate^10^.

Experimental studies have used EPR to evaluate the opening degree of the NBD dimer according to the nucleotide state^11,12,25^. In most cases, the measurements were done using pairs of residues in the helical domains, in contrast with our measurements using the C-α atoms of the ATP binding motifs. Additionally, these studies use ADP in the post-hydrolysis state rather than ADP.Pi, as we did. Therefore, the results on Fig. 2 are not directly comparable with the experimental results.

#### On the transmembrane domains and MalE

In all ABC transporters, ATP hydrolysis is known to cause conformational changes, which result is different outcomes, namely for substrate release or to reset the transporter to the starting point after a transport cycle^26,27^. In the case of the maltose transporter, ATP hydrolysis triggers conformational changes that result in the reorientation of the transmembrane domains, creating an inward-facing state allowing the release of the substrate and MalE disengagement^9,25^. It is hypothesized that the closed, outward-facing form of the maltose importer is a short-lived high-energy catalytic intermediate^5^. In fact, upon MalE binding and NBD closing, ATP hydrolysis is highly stimulated^9,11,13^.

**Figure 3:**
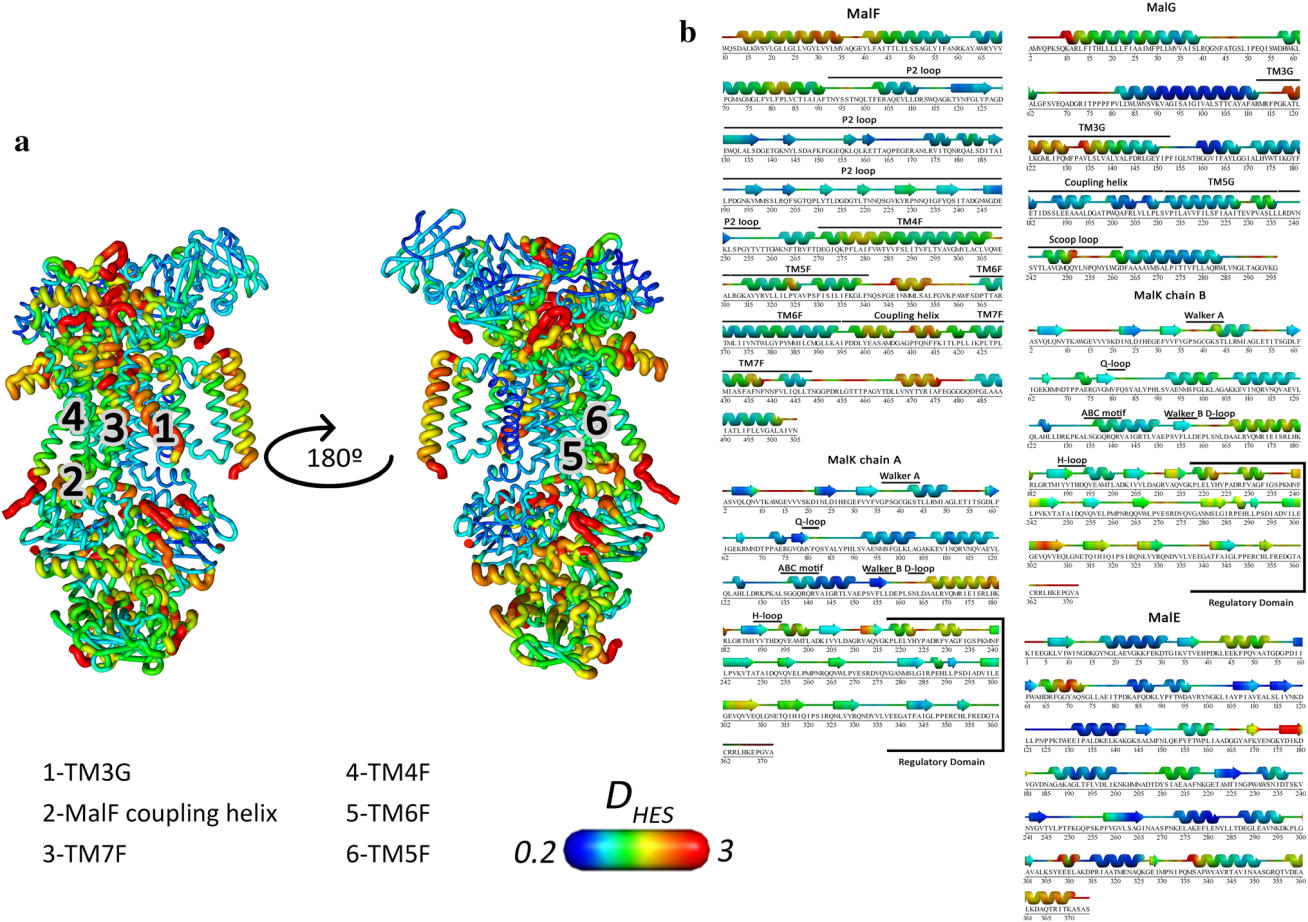
Mapping of the harmonic ensemble similarity (D_HES_) between the ATP and ADP states. D_HES_ was calculated for each residue, using the C-α atoms. The last 50 ns of each trajectory were used for this analysis, and the values presented result from the average of cross-replicate comparisons. The scale below indicates the magnitude of the changes in D_HES_ observed, in which the dark blue corresponds to the minimum values observed, indicating increased similarity while the red corresponds to the maximum values observed, indicating increased dissimilarity. (**a**) D_HES_ values mapped on the protein structure. The structure in the left represents the “front view” of the transporter, while the one on the right represents the “rear view”, accomplished by a 180° rotation. Relevant regions are marked with numbers: 1-Transmembrane helix 3 MalG (TM3G), 2-MalF coupling helix, 3-Transmembrane helix 7 MalF (TM7F), 4- Transmembrane helix 4 MalF (TM4F), 5-Transmembrane helix 6 MalF (TM6F), 6-Transmembrane helix 5 (TM5F). (**b**) Mapping of D_HES_ between the ATP and ADP states, on the sequence of each chain. The 3D structure map was obtained with PDBsum^68^.

In order to assess the structural impact of hydrolysis in the full-length complex, the harmonic ensemble similarity (D_HES_) between ATP and ADP states was calculated for each residue, using the C-α atoms. Figure 3 shows D_HES_ calculated using the last 50 ns of the ATP and ADP simulations. ADP hydrolysis has led to significant differences in the distributions sampled by most residues throughout the complex. In the NBD dimer, there are key regions affected by hydrolysis, such as the D-loops, the Walker B motifs as well as the catalytic motifs, the Walker A and ABC sequences (Fig. 3). The α-helical domains also display significant changes, that are transmitted to regions of the regulatory domains nearby (Fig. 3a, marked with the number 2). Nonetheless, the MalK chain B is clearly more affected by ATP hydrolysis, showing higher D_HES_ values. Interestingly, this NBD contacts with the MalF coupling helix that displays significant conformational changes that are transmitted to the neighbouring helices 4, 5, 6 and 7 from MalF (TM4F, TM5F, TM6F and TM7F) (Fig. 3b, numbers 3 to 6 in the Fig. 3a). The P2 loop does not show significant changes. The conformational changes are transmitted to MalG through TM7F reaching transmembrane helix 3 in MalG (TM3G) (Fig. 3b, number 2 in Fig. 3a). This is remarkable since MalG transmembrane helices are fairly rigid in comparison with MalF. Nonetheless, the residues at the end of transmembrane helix 5 of MalG show high D_HES_ values. Furthermore, the MalG scoop loop that plays a role in maltose transfer from MalE to the transmembrane domains also shows high *D*_*HES*_ values (Fig. 3b). Other external loops from MalG and MalF that contact with MalE externally also show large variation (Fig. 3a). Regarding MalE, significant differences are observed, not only in the regions in direct contact with the transmembrane domains, but also in the C-lobe (residues 163–187 and 326 to 374 in Fig. 3b).

Two dimensional PCA analysis of the all the simulated states allowed us to obtain two main clusters of basins: one containing only Apo conformations and other with mixed ATP/ADP content (Fig. S9 and S10). Therefore, the Apo state can be fully separated from the ATP and ADP states (Fig. S10). Regarding the cluster of basins belonging to the ATP and ADP states, these two states cannot be distinguished so easily, since they show overlap in some of the basins (Fig. S10), but in different degrees (Table S3). Nevertheless, it is possible to identify characteristic basins of each state, which show a markedly ATP or ADP character, containing a larger proportion of conformations within that basin (Table S3). By analysing the structures corresponding to the maximum probability on each of the most characteristic basins, it is possible to get a more detailed view on the conformational changes suffered by the most relevant regions above mentioned. Even though this analysis focuses on limited zones of the whole conformational space, the comparison between the characteristic structures of these two basins allows us to identify what is markedly different between the ATP and ADP states, and their difference from the Apo state. This is what is shown in figure S11. The Apo state shows a unique conformation that is clearly distinguishable from the ATP and ADP ones, in which the periplasmic portion of TM7F, moves to the center of the pore causing constriction in the region containing the periplasmic gate (Figs. S11B and S11F). On the other hand, TM6F moves away from the pore center (Fig. S11C), while the MalF coupling helix (Fig. S11A) and TM4F adopt a unique position different from the other states (Fig. S11D). Focusing now on the ATP and ADP states, we can see that ATP hydrolysis results on a shift of the MalF coupling helix (Fig. S11A) and TM6F moves towards the NBD direction (Fig. S11C), while TM7F is shifted in the opposite direction and it moves slightly to the exterior of the pore (Fig. S11B). On the other hand, TM4F shifts towards the MalE direction (Fig. S11D). ATP hydrolysis also prompts a displacement of the transmembrane helix 5F (TM5F) towards the periplasmic direction (Fig. S11E). Nonetheless, the most remarkable alterations happen in the bottom of the pore, where there is a major lateral displacement of TM3G (Fig. S11F). The TM5F and TM7F helices also suffer lateral displacements in this region (Fig. S11F), also causing pore constriction, similarly to the Apo state.

Wen et al. suggested that the NBD-TMD coupling is done by a network of contacts that arises from the ABC motif, goes to the helical domains, followed by the Q-loop, ending on the coupling helices^28^. In fact, our results confirm these observations, by showing considerable differences in the ensembles sampled by the residues in this region.

The active site 2, that shows a larger degree of opening (see Fig. 2), is composed by the chain A Walker A motif and by the chain B ABC motif. Upon hydrolysis, the residues of the ABC motif show higher dissimilarities on the sampled ensembles, than the Walker A residues. Considering that the MalK chain B is connected with MalF and that MalF shows larger D_HES_ on key regions throughout the complex, it is possible to hypothesize a mechanism for signal transmission, from the NBDs to the remaining protein, in which the higher motion of the ABC motif contributes for spreading the signal across MalF and the remaining complex. This stronger coupling with MalF is also corroborated by cryo-EM structures in which an asymmetric NBD dimer was observed, with MalF shifted from the center of the complex^10^. We have also observed novel conformational changes upon hydrolysis in the MalG subunit, such as the displacement of the transmembrane helix 3 (TM3G) at the periplasmic end of the pore, contributing for pore narrowing in that region.

In this way, ATP hydrolysis in the NBDs impacts the entire protein complex. Conformational changes in the transmembrane helices lead to variations in the pore properties, such as the pore radius and residue conformation.

**Figure 4:**
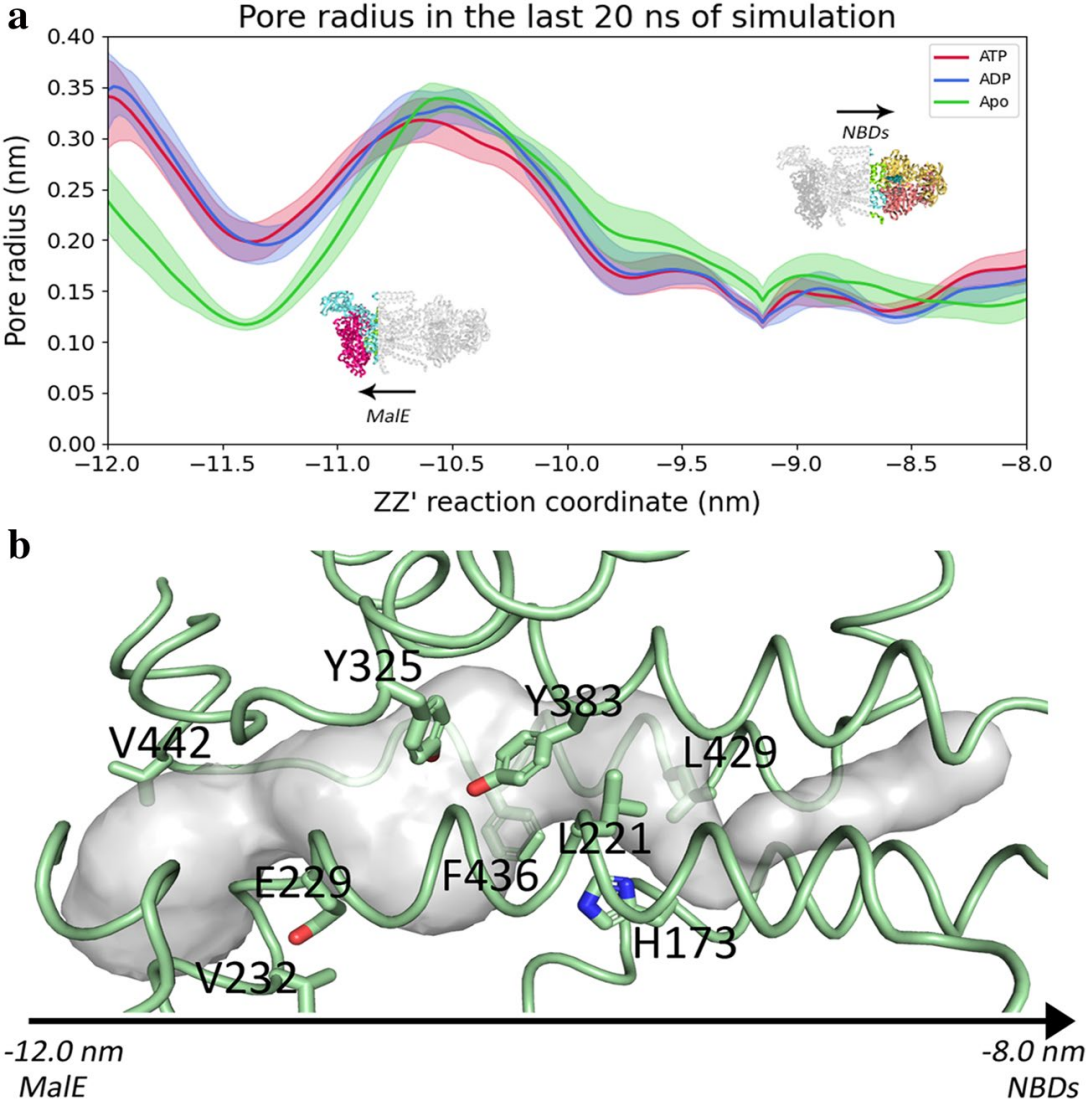
Pore radius across the channel. (**a**) Pore radius across the channel in the last 20 ns of simulation. The ATP state is represented in red, while the ADP one in blue. The observed values are the average for each state and the error bars represent the 95% confidence interval obtained by bootstrapping. Larger values of the zz’ reaction coordinate indicate a path towards the intracellular medium, while lower values indicate the direction towards MalE and the periplasmic medium. (**b**) Pore representation with relevant residues. The pore surface was generated using HOLE^65^. The residues are displayed in sticks, the pore volume as surface and the protein backbone as ribbon. The bottom of the pore heads towards the periplasmic direction.

Figure 4a shows the pore radius profile on the last 20 ns of simulation in all the simulated states. Overall, the transmembrane pore is constituted by a pear-shaped cavity, wider at the bottom (near the periplasmic gate) becoming increasingly narrowed as it progresses towards the cytoplasm (Fig. 4b).

The region around −11.5 nm corresponds to the constriction point caused by the periplasmic gate residues (V442 and V232) and it is a local minimum in the ATP and ADP states and a global minimum in the Apo state. Downstream from this region, there is the MalE binding site. The Apo state is rather distinct of the other two states, mostly the part towards the periplasmic gate. These differences reflect the initial conformation of the protein in the Apo state, which has the transmembrane helices much more packed, leading to smaller radius. Despite the great similarity of the ATP and ADP profiles, hydrolysis causes a global change on the radius profile, leading to a further decrease of the radius in the MalE binding site (below −11.5 nm) and in its vicinity up until −11.0 nm. On the other hand, there is a concomitant increase of the radius above this region (Fig. 4). Nonetheless, the most striking difference upon ATP hydrolysis is the increase of the pore radius that occurs in the −10.8 to −10.2 nm range, but there are still differences up until −9.5 nm, in which the error shading of the ADP state surpasses the ATP state in the upper limit. This region comprises the maltose binding site and it enclosed by E229 in its periplasmic end, and by Y383 and H137 in the cytoplasmic gate region. The aromatic residues Y325 and F436 are contained in this region. The minimum observed at −9.2 corresponds to the cytoplasmic gate residues (L429 and L221) and it is a main constriction point of the pore. The values upstream this cytoplasmic gate reflect the path until reaching the coupling helices. Overall, ATP hydrolysis leads to the compression of the pore near the periplasmic end, confirming the observations of the PCA analysis, in which TM3G, TM7F and TM5F move towards the pore center (Fig. S11F). A concomitant increase of the region containing the binding site (from −10.8 to −10.2 nm region) along with a marked increase of the region spanning the range from −10.2 nm to −9.5 nm, reflects the motions of the MalF coupling helix, TM7F, TM6F and TM5F on this portion of the pore (Fig. S11A, S11B, S11C and S11D).

The last 20 ns of simulation were chosen to represent this property, because it is the timeframe that allows to observe the maximum differences. The similarity of the Apo radius profile with the ATP and ADP profiles in the region towards the NBDs (mainly from −10.5 nm onwards) is due to the sudden closure observed in the beginning of the simulations (Fig. S8). This same behaviour was previously described by Weng et al.^14^, who performed metadynamics simulations on the Apo structure. Nevertheless, it should be noted that the displayed profiles are averages of multiple conformations and may not reflect the conformational diversity of each state.

Despite the several models for MalE action throughout the transport cycle^9,11,12,29,30^, it can be speculated that the effect of ATP hydrolysis, along with the progressive conformational changes in MalE, results in an increasingly inward-facing state. This leads to an increase of the pore radius, facilitating substrate diffusion upwards and eventual exchange with the nucleotides. In fact, EPR data confirm that the nucleotide state in MalK deeply influences the coupling of P2-loop, with MalE eventually altering its conformation^30^, and that in the presence of ADP, the MalE N-lobe adopts an unique conformation^12^, while the MalE C-lobe is more disordered. This can potentially shape the TM helices into the inward-facing state in a concerted action with the NBDs. In addition, there are contradictory experimental data concerning the affinity of the open and closed MalE to the transmembrane complex^9,12^.

Nonetheless, with MalE always bound, the unidirectionality of the transport is safeguarded. It may also be possible that ADP or phosphate exit are required to further enhance these transformations.

**Figure 5:**
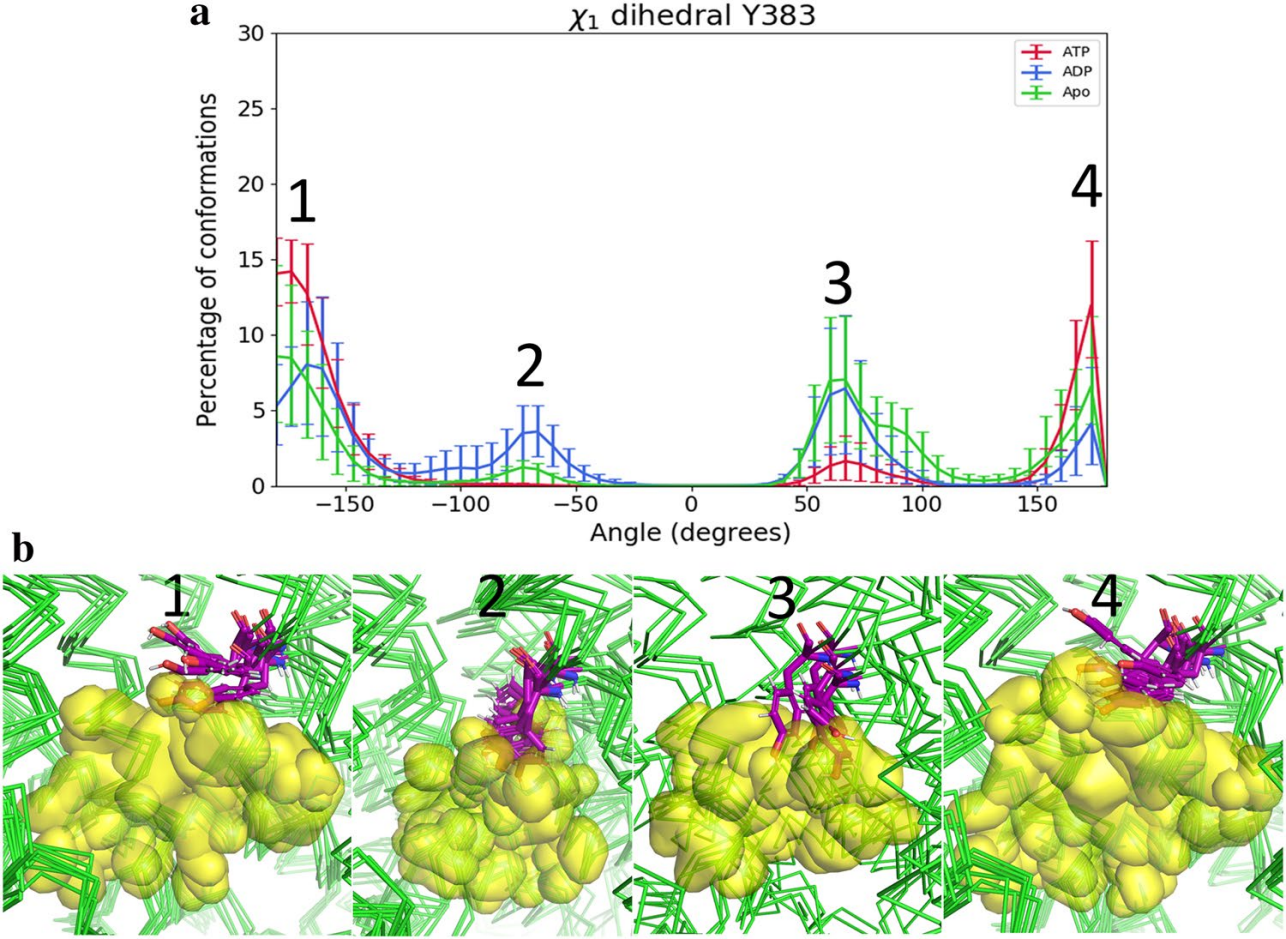
Behaviour of ${\upchi }_{1}$ dihedral values for Y383 for all states. (**a**) Average ${\upchi }_{1}$ dihedral values for Y383 in each state. The ${\upchi }_{1}$ reflects the rotation of the first bond between the C-α atom and the aryl ring. ATP is represented in red, ADP in blue and the Apo state in green. The main populations observed are indicated with numbers from 1 to 4. The error bars represent the 95% confidence interval obtained by bootstrapping. (**b**) Examples of representative conformations for each main population observed. The protein is coloured in green, and Y383 is represented in purple sticks and according to the atom type, while maltose as a density surface in yellow.

Regarding the behaviour of the pore residues, the most noteworthy residue is Y383, not only because of its position, located below the cytoplasmic gate, but also because its conformation changes drastically upon the nucleotide state of the protein, as shown in Fig. 5. When measuring the ${\chi }_{1}$ tyrosine dihedral, it is possible to differentiate four populations (Fig. 5a): a first population with ${\chi }_{1}$ values between −180° and −150°. This population is most abundant before hydrolysis and corresponds to conformation in which the hydroxyphenyl ring is adopts an orientation perpendicular to the pore axis, preventing maltose diffusion from this point upwards as seen in Fig. 5b. Additionally, the last population with ${\chi }_{1}$ values between 150° and 180° also displays a similar behaviour and it is also most abundant on the ATP state. Upon ATP hydrolysis two other populations arise: one with ${\chi }_{1}$ values between −100° and −50°, most abundant in the ADP state and other with ${\chi }_{1}$ values between 50° and 100°, most abundant in the Apo, followed by the ADP state. This last population is more energetically accessible than the previous one, as seen by the higher percentage of conformations that occupies the same space, being even accessible by the ATP state. In these two populations Y383 adopts a conformation parallel to the pore axis, with the sidechain being away from the pore centre. This behaviour may be a consequence of the conformational changes described above, since Y383 is located in TM6F of MalF, which is one of the most affected helices, as seen before. Mutation of Y383 to a serine residue led to lower growth rates using maltose, but not maltoheptatose, leading to a possible change of the substrate specificity^31^. Therefore, Y383 is a key residue that controls substrate efflux and involved substrate specificity. In this way, it can be considered a first gatekeeper before the periplasmic gate.

### Effect of hydrolysis and nucleotide exit on maltose diffusion and binding

**Figure 6:**
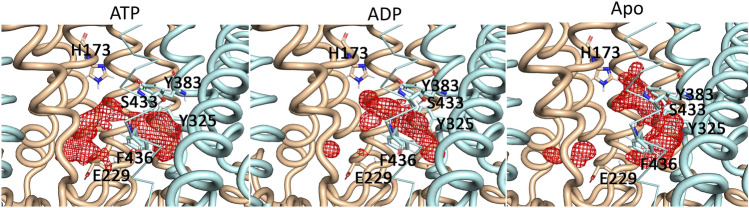
Maltose binding in the transmembrane domains. The isosurface was build using the center-of-mass positions of maltose. MalG is represented in beige, while MalF in light blue. Key residues that interact with maltose are represented in sticks. The red mesh represents the distribution of the maltose center-of-mass.

As a consequence of the helical displacement upon hydrolysis and nucleotide exit, the pore volume is increased in some regions, which culminates in increased maltose vertical diffusion. Figure 6 shows the diffusion maps of the maltose center-of-mass for the three states. It is possible to observe two main binding sites. Prior to hydrolysis it is possible to observe that a large portion of the density is in the vicinity of MalG (at the left side of the picture). This binding spot is constituted by hydrophilic residues, such as the E229, Y166, S135, H173 and N129, along with hydrophilic mainchain groups. In addition to this spot, a significant amount of density is observed in the vicinity of MalF. In this spot, maltose is surrounded by a cluster of aromatic residues, formed by F436, Y325, Y383, along with sidechains from polar residues, mainly asparagine and serine residues. These interactions are constant in all the simulated states (Figs. S12 and S13) and are also present in crystal structures that were the starting points of this work. The importance of these hydrophobic interactions is assessed by the fact that mutations in F436 and Y383 reduce transport activity by over 90% in comparison to the wild-type, and the mutation Y325S retains only 52% of the transport activity^31,32^. Figure 6 shows that this pocket in MalF becomes increasingly populated and expands upwards after hydrolysis and nucleotide exit. The pre-hydrolysis and pre-translocation X-ray structures show maltose bound to this pocket in MalF. However, our simulations revealed the existence of an additional pocket in MalG in the pre-hydrolysis state, with a more reduced occupancy in the post-hydrolysis and nucleotide-free states.

The existence of this additional pocket is corroborated by the crystal structure of MalFGK_2_E with maltoheptatose bound in which at least one of the glucosyl units is pending towards MalG (in this structure, the other unit lacks electron density)^24^.

Maltose seems to make a similar number of hydrogen bonds with MalF and MalG in either of the ATP and ADP states, displaying a slight preference for MalF in the Apo state (Fig. S14). The residues which make the most hydrogen bonds belong to MalF and are located within and nearby the cluster of aromatic residues previously mentioned—Y383, Y325, N376 and S433, along with H173 that belongs to MalG and is located upstream to this spot (Fig. 6 and Fig. S12). Maltose also interacts with E229 (Fig.S14), which is part of MalG and is located in the vicinity of the scoop loop towards the periplasmic side. Interestingly, this interaction is highly dependent of the nucleotide state, being most frequent in the ATP state, followed by the ADP and even lower in the absence of nucleotide. The movement of the transmembrane helices, which increases the pore volume and vertical diffusion, can explain these differences, namely changes in helices TM4F, TM5F, TM6F, TM3G and TM5G, which contain key residues that influence substrate transport such as the periplasmic gate residues V442 and V232, the cytoplasmic gate residues L429 and L221, and Y383. Mutagenesis studies show that deletion of E229 leads to the failure in complex assembly and maltose transport^33^. It is possible to hypothesize that it may facilitate maltose diffusion from MalE to the transmembrane domains, or even prevent maltose diffusion in the opposite direction towards MalE.

Nonetheless, the most frequent interactions performed by maltose are with water molecules, consequence of the high solvation of the pore (Fig. S15).

### Effect of hydrolysis and nucleotide exit on maltose translocation

**Figure 7:**
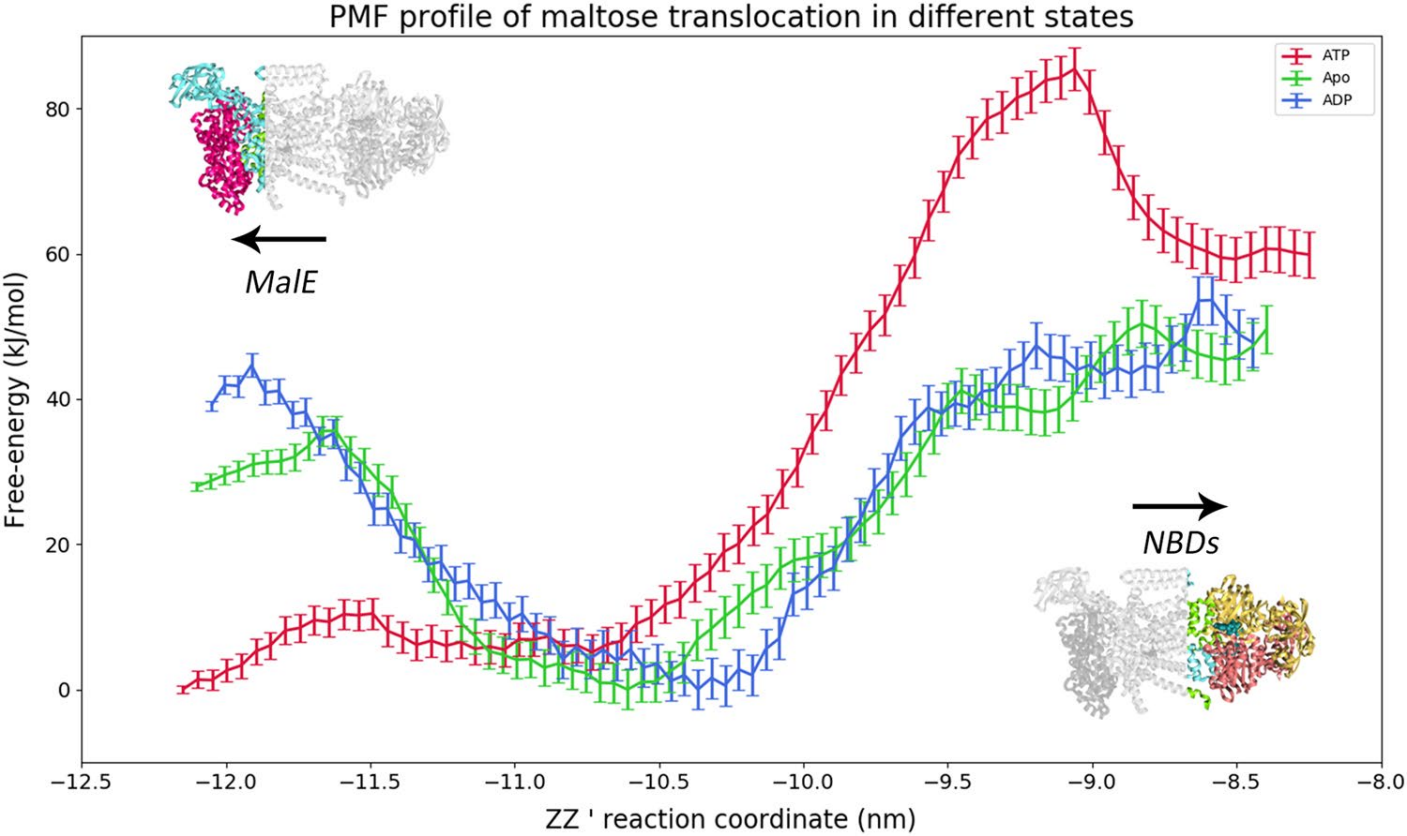
Potential of mean force (PMF) profiles for the ATP, ADP and Apo states. The ATP profile is represented in red, the ADP profile in dark blue and the Apo profile in green. The error bars correspond to a 95% confidence interval at each point, assuming that the energy values in each bin follow a normal distribution.

The effect of ATP hydrolysis and nucleotide absence on maltose translocation was studied using both pulling and umbrella sampling MD simulations. Conformations were extracted from the equilibrium MD simulations at different times and pulling was performed in order to generate multiple maltose conformations across the channel. The initial positions of maltose used in each window are described in tables S4 to S6. Maltose was in various initial positions across the channel in the different frames extracted. Figure 7 shows the potential of mean force (PMF) profiles obtained for the several states. The lower reaction coordinate values point towards the periplasmic side of the protein, where MalE is bound, while the higher values point towards the cytoplasmic side, where the NBDs are located. The histograms used to make the PMF profiles, along with the convergence tests can be found in Figs. S16 to S24. The maltose conformations sampled spanned the lowest point possible towards the periplasmic side, up until reaching the vicinity of the NBDs. The high free-energy values, from 20 to 80 kJ/mol, possibly result not only from structural constraints, but also from instabilities generated by the pulling process, resulting in an artificial biasing of the energy values. We also recognize that our sampling is not perfect leading to an additional roughness of the profiles. Therefore, all the conclusions deducted henceforth will be of a strictly qualitative nature.

Overall, the profiles span around 3 nm, from −12 nm to ≈8.3 nm. The profiles show a local maximum around −12 nm to −11.5 nm, followed a global minimum in the region of −11 nm to −10 nm. This is followed by an increase of the free-energy towards the cytoplasmic side, until reaching the NBD level. The behaviour of these PMF profiles is inversely correlated with the pore radius behaviour, across states. Nonetheless, they are not tightly correlated, because the pore radius represented in Fig. 4a, is averaged over the simulation time, while the PMF profiles were obtained by extracting individual conformations from the MD simulations, upon which pulling, and umbrella sampling simulations were performed. Figure S25 shows the average pore radius obtained in umbrella sampling simulations and it is more correlated with the PMF profiles.

The ATP PMF profile shows a local maximum at around −11.5 nm, corresponding narrowing of the pore in between the periplasmic gate valine residues: V442 and V232. At this point, maltose is in a predominantly vertical conformation, as calculated by the angle between the z-axis and the vector that links the most distant carbon atoms in the maltose rings (Fig.S26). The maltose conformation contributes to account for the high free-energy values near the periplasmic gate region. This region of the pore is considerably tighter as seen by the low pore radius shown in Fig. 4. Therefore, when maltose is in a horizontal conformation, its presence will not be energetically favourable in that portion of the channel. The vertical conformation will decrease the steric clashes with the protein residues. From −11.5 nm and under, maltose is below the periplasmic gate, interacting with the scoop loop from MalG, as well as some MalE residues, such as K46. The fact that maltose is able to adopt a horizontal conformation, as seen by the highly populated bins between 80° and 120° in Fig. S27 (ATP state) is a consequence of the larger pore radius observed in this region. This leads to lower free-energy values prior to the local maximum. This local maximum is located downstream at −11.5 nm in the ADP and Apo profiles. This is a consequence of the different conformations assumed by the protein in the different states.

In fact, in all the simulated states, maltose tends to adopt a horizontal conformation near the periplasmic gate, being later reoriented to cross the periplasmic gate (Fig. S23). This location corresponds to a local maximum in all PMF profiles. Interestingly, this energy barrier is lower prior to ATP hydrolysis, suggesting increased diffusion probability towards MalE. Therefore, ATP hydrolysis enhances the irreversibility of the transport process, being a consequence of the motion of the transmembrane helices. When maltose is between the periplasmic gate residues, it starts to interact with the E229 as well as other MalF residues, such as S329 and N440. Nonetheless, the visualization of the trajectories shows that E229 plays a key role in redirecting and reorienting maltose towards the maltose binding site on MalF, which constitutes the energy minimum observed in all the profiles. This binding site is the same described above, and the one observed experimentally in the X-ray structures.

**Figure 8:**
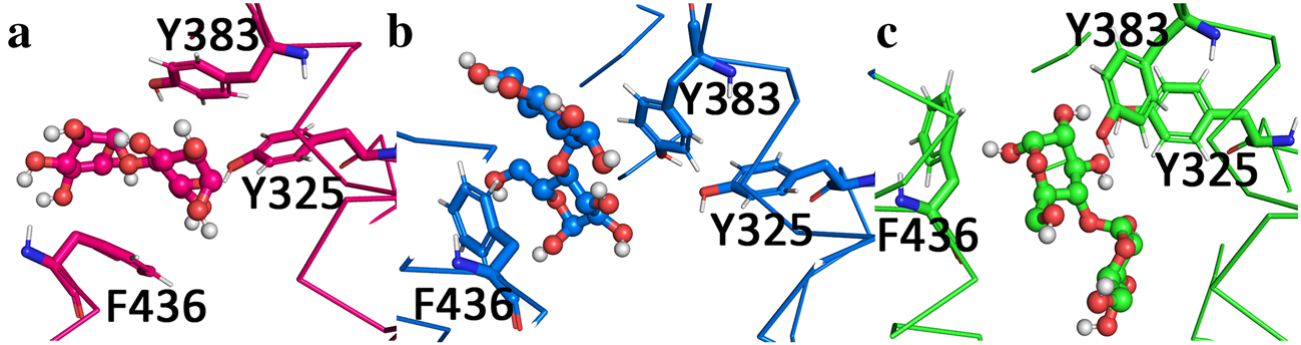
Maltose binding pocket with maltose bound, surrounded by key residues. (**a**) Maltose binding pocket in the ATP state, maltose is represented in sticks and spheres, while the residues are portrayed in sticks only. (**b**) Maltose binding pocket in the ADP state, maltose is represented in sticks and spheres, while the residues are portrayed in sticks only. (**c**) Maltose binding pocket in the Apo state, represented in blue and green, respectively. The relevant aminoacids are represented in sticks, while maltose is represented in spheres and sticks.

The global minimum observed in the PMF profiles, around −10.5 nm corresponds to the maltose binding site. The maltose binding site in MalF is delimited by three aromatic residues: Y383, Y325 and F436 as seen in Fig. 8. Maltose establishes hydrophobic interactions with these residues as well as hydrogen bonds with the tyrosine hydroxyl groups. The residues in the pocket change conformation upon ATP hydrolysis and nucleotide exit (Fig. 8).

The PMF profiles confirm the importance of Y383 as a gatekeeper. By changing its conformation upon hydrolysis, allows maltose to adopt a vertical conformation and removes any vertical constraints to diffusion, while the remaining interactions become weaker, such as the case of F436 and Y325 (Fig. 8b,c). This vertical conformation seems to be associated with lower energies, as a result of lower steric strains (Fig. S27), being able to cross the pore more easily. The predominant conformation of maltose in the Apo and ADP profiles is in a vertical orientation (aligned with the pore axis) (Fig. S26 and S27).

E229 not only guides maltose towards the hydrophobic pocket, but it also guides maltose out of this pocket, as maltose leaves it by making hydrogen bonds with this acidic group. In the region of −9.5 nm to −9 nm, it is possible to find an energy maximum which corresponds to the cytoplasmic gate, and this maximum results not only from the presence of the bulky leucine residues, but also because it is the point of maximum constriction in the pore, i.e., minimal radius (Fig. S26). Closer to the cytoplasmic gate, H173 is also key for ensuring a proper vertical orientation, from the pore center up to the vicinity of the cytoplasmic gate. There are also additional interactions with T176. The vertical conformation of maltose allows it to cross the cytoplasmic gate and diffuse in a rectilinear way towards the NBD vicinity.

In contrast, in the ATP state, maltose reaches the cytoplasmic gate in a horizontal conformation. Due to the low radius in this region and above, maltose has the tendency to diffuse laterally towards the direction of TM5F and TM6F, reaching the MalF coupling helix. Interestingly, there is a cluster of hydrophobic residues in this region, mainly valine and leucine residues, creating a highly unfavourable environment for the residence of maltose in this place, resulting in a peak of free-energy from −9.2 nm to −9 nm, approximately. The subsequent decrease corresponds to the maltose diffusion towards the NBDs and to the middle of the pore.

After the cytoplasmic gate, the region between −9 and −8.5 nm is lined by hydrophobic residues, namely isoleucine, leucine, valine and tyrosine residues. Maltose is still able to perform a few hydrogen bonds with backbone atoms and a few polar residues (Y180, T176, T290), but the hydrophobic character of this region may well contribute to accelerate maltose exit towards the cytoplasm. The ADP profile also shows a local maximum at −8.5 nm that corresponds to an horizonal conformation of maltose, at the end of the TMDs and beneath the NBD dimer. All the PMF profiles end in the terminus of the TMDs, beneath the NBDs.

The ADP and Apo PMF profiles show substantially lower energies from the maltose binding pocket onwards, reflecting all conformational changes on the membrane helices and key residues, such as Y383, which facilitate translocation from that point onwards.

Interestingly, despite the pore constriction in the direction towards the NBDs, maltose is highly solvated, but the solvation degree slightly decreases upon hydrolysis and nucleotide exit (Fig. S27). Most hydrogen bonds at the bottom of the protein pore are performed with MalF, and as maltose diffuses upwards, the number of these interactions with MalF decreases. In the ATP state, this is accompanied by a decrease in the interactions with MalG, but this is compensated by a higher amount of hydrogen bonds with water molecules. On the other hand, in the ADP and Apo states there is an increase of the hydrogen bonds with MalG, while the solvation sphere of maltose is smaller. As expected, all interactions decrease when reaching the cytoplasmic gate, due to the constriction in this region. In fact, the rise of the free-energy along the reaction coordinate is highly correlated with the progressive decrease of the pore radius (Fig. 4 and S25).

The free-energy differences observed in the PMF profiles are correlated with the pore radius (Fig. 7 and Fig.S25). In the periplasmic side of the profile (between −12.0 and −11.0 nm), the pore radius decreases upon hydrolysis and further decreases upon nucleotide exit, which coincides with an increase on free-energy observed in PMF profiles, meaning that the maltose has little tendency to remain in this portion of the pore. This is a consequence of the conformational changes observed in the transmembrane helices TM7F, TM5F, TM6F and TM3G that lead to the constriction of this region (Figs. 3 and S11). Nonetheless, in the binding site region the free-energy of translocation is similar in both ADP and Apo states, despite the ADP pore radius being similar to the ATP. We attribute this difference to the change in maltose orientation to the vertical orientation (Fig.S27). From the maltose binding site towards the NBDs, the free-energy of translocation becomes similar in both ADP and Apo states, reflecting the similarity in the pore radii of both states (Fig.S25). These alterations cause the constriction of the pore towards MalE while enlarging the pore in the opposite direction. The importance of the conformational changes in the transmembrane helices becomes evident since these key residues are located in the above referred helices. Y383 is located in TM7F, while F436 and L429 (cytoplasmic gate) and V442 (periplasmic gate) are located in TM6F. The residue V232 from the periplasmic gate belongs to TM5G, which was also affected by hydrolysis.

The PMF profiles, along with the structural information obtained in the umbrella sampling simulations, provide important clues on the transport mechanism. It is possible to observe that upon ATP hydrolysis and posterior nucleotide exit, the transport process becomes increasingly favourable from the energetic viewpoint. Nonetheless, the Apo PMF profile is quite similar to the ADP one, suggesting that hydrolysis is the main drive to lower the translocation energy, rather than nucleotide release, as seen by the similarity of PMFs along the reaction coordinate, but mainly at the top near the NBDs. In fact, this goes in agreement with the Cryo-EM data that shows that NBDs, in the absence of nucleotide, are mainly in a semi-closed state, while maltose and MalE are still present in the complex, suggesting that maltose exit is one of the last steps before transporter reset^11^. Further kinetic data^12^ shows that phosphate release is the limiting step of the transport cycle and ADP exit can enhance NBD opening. The authors also suggest that the release of Pi is accelerated by the presence of maltose. Therefore, it is possible to assume that phosphate is released prior to maltose.

This decrease in free-energy upon hydrolysis and nucleotide release happens due to conformational changes triggered by both processes in the transmembrane helices. The closure of transmembrane cavity starting from the periplasmic side, with the concomitant opening of the pore on the cytoplasmic side, happens as a result of the motions in transmembrane helices TM7F, TM5F, TM6F and TM3G (Fig. 3). These concerted motions lead to an increase of the free-energy values in the periplasmic region (−12 to −10.5 nm) with the simultaneous decrease on the cytoplasmic side (from −10.5 nm onwards). This process somewhat resembles the peristaltic transport mechanism suggested for the type II importer BtuCD, in which the sequential closing of the periplasmic gate, followed by the opening of the cytoplasmic gate, creates a peristaltic movement, allowing substrate translocation^34^. However, the structures of type II importers are substantially more rigid, and the existing mechanisms reject allosteric coupling between the NBDs and SBP, which has already been proven to happen in the maltose importer by a plethora of experimental data^9,11–13,30^.

Considering that our results show similar energetic behaviours for substrate translocation with ADP and in the absence of nucleotide, it is possible to question whether MalE has any influence on the transport process. Data from X-ray structures^7,24^, EPR^11,12^ and cryo-EM^10^ experiments support the hypothesis that MalE remains bound throughout the entire conformational cycle. Furthermore, it is also hypothesized that MalE may have a role in the irreversibility of the transport. Our simulations did not show any significant conformational changes that could indicate MalE unbinding. Additionally, different transport models^9^ defend that MalE is bound in different binding states – open or closed throughout the cycle. Previous reports using cryo-EM structures of MalFGK_2_E^10^, obtained with maltose and MalE bound, showed that in 32% of the particles, MalE detached, raising the possibility that MalE unbinding precedes maltose release. Therefore, it is possible that the weakening of interactions of MalE with the membrane complex results in the increase of NBD opening, further decreasing the free-energy barrier, facilitating maltose exit.

Based in this information above presented, we suggest a possible model for MalFGK_2_E function, in which ATP hydrolysis leads to NBD opening causing rearrangements of the transmembrane helices stimulating vertical diffusion. Yet, further diffusion towards the cytoplasm and eventual substrate release might require the rearrangement of MalE, resulting in the weakening of MalE interactions with the transmembrane complex and increasing the probability of diffusion outside the complex.

The mechanisms unveiled for the MalFGK_2_E importer slightly resemble other sugar transporters, such as the ones that belong to the major facilitator superfamily (MFS). In a similar way to ABC transporters, MFS proteins alternate between the inward and outward facing conformations, but not powered by ATP hydrolysis^35^. In the GLUT1 transporter, the conversion from the outward to the inward-facing state leads to rearrangements of the transmembrane helices and other loops. The pore closes from the extracellular side, leading to the diffusion of glucose from the sugar binding site to an intracellular gate. The resulting PMF of translocation shows an uphill behaviour towards the exit with a local minimum corresponding to a transient sugar binding site constituted by hydrophobic residues^36^. The GLUT4 transporter also possesses aromatic residues and a glucose binding site that aid in the reorientation and positioning of glucose in the pore, in order to interact with the appropriate polar groups. The main energetic barriers were related with hydrogen bond breaking/formation upon entry and exit, with small oscillations along the channel, mainly related with reorientation^37^.

When comparing the MalFGK_2_E importer mechanism with the sugar channel LamB located in the *E.coli* outer membrane, responsible for maltose intake to the periplasmic space, significant functional differences arise. In LamB, translocation is driven, not only by molecular interactions with hydrophobic residues (the so-called “greasy slide”), but also by hydrogen bonds with polar residues. The asymmetric distribution of residues in the pore also contributes to enhance substrate entry from the extracellular side^38^, which allows substrate diffusion in a screw-like manner, interacting with the hydrophobic residues and polar residues, in which the latter compensate for the dehydration process^39^. In this way, it is possible to have a passive translocation process with little conformational changes in the protein. Translocation studies show a PMF profile with the main barriers located at the entry and exit of the channel that correspond to forming and breaking hydrogen bonds with the protein and the solvent^40^. In contrast, our simulations show that in the MalFGK_2_E maltose importer, ATP hydrolysis triggers motion of the helices in order to stimulate upwards diffusion, but maltose is still reasonably solvated and seldom interacts with the hydrophobic residues along the pore.

## Conclusions

In this work, we have performed molecular dynamics simulations of the *E.coli* MalFGK_2_E importer in the pre and post hydrolysis state, as well as in the absence of nucleotide, with the goal of assessing the effects of ATP hydrolysis and nucleotide exit on the translocation of maltose.

We concluded that ATP hydrolysis triggers a series of conformational changes in the protein complex, starting by pocket opening of the active site, and spreading these conformational changes through the transmembrane domains reaching MalE. Hydrolysis affects critical transmembrane helices that conduct to pore constriction at the periplasmic side, while enlarging the regions towards the cytoplasmic side, leading to increased maltose diffusion towards the NBDs. Y383 was identified as a novel gatekeeper prior to the periplasmic gate, and key to allow substrate diffusion. Additionally, a novel binding spot in MalG was found, with maximum occupation in the ATP state. The post-hydrolysis state showed similar properties to the state without nucleotides.

The PMF profiles of maltose translocation show that hydrolysis significantly lowers the energetic barriers of substrate diffusion towards the intracellular medium, with a concomitant increase of the energy in the opposite direction. Therefore, ATP hydrolysis considerably contributes for the irreversibility of the transport process.

Our data suggest that the maltose binding pocket may play a role in substrate reorientation from the periplasmic side onto the cytoplasmic side. Other key residues, such as E229, Y383 and H173 assist this task.

Interestingly, nucleotide exit does not lead to significant differences in maltose permeation free-energy profile, when comparing with the ADP state. In this way, it might be necessary a weakening in the MalE interaction with the rest of the complex in order to increase NBD separation and further TMDs transformations, leading to a further decrease of the energetic barriers. Further investigations are required to fully understand this phenomenon.

## Methods

### System setup

In order to achieve the goals previously stated in the introduction, we simulated three relevant states; (i) the pre-hydrolysis state with ATP bound in the NBDs, to which the starting point was the pre-hydrolysis structure crystallized with ANP-PNP (PDB code: 3RLF)^41^; (ii) The post-hydrolysis state was generated from this state as explained on Sect. 2.3; (iii) The Apo state, mimicking the nucleotide exit was simulated using the pre-translocation state (PDB code: 3PV0)^7^. We did this because there is no available X-ray structure explicitly reflecting the nucleotide exit state, but experimental EPR data shows that the post-hydrolysis state is highly similar to the pre-translocation structure ^11,12,25^. The main similarities comprise the semi-open NBD dimer^11,12,25^ and MalE binding to the complex^9,12^. In fact, experimental EPR and Cryo-EM show that MalE induces closure of the NBD dimer and stabilizes a semi-open NBD conformation in the absence of nucleotide, such as the one observed in this structure^10–12^.

The missing segments were rebuilt using MODELLER version 9.6^42^. All structures were rebuilt in order to have the same number of residues, with the goal of increasing the structural similarity. In the pre-translocation structure (PDB code: 3PV0), the reconstructed segments were: residues 371 to 374 in MalE, residues 10 to 28, 242 to 248, 504 to 505 in MalF, and the segment 280 to 296 in MalG. Additionally, the dissulfide bridge between residues 69 and 337 in MalE, which was engineered for crystallisation, was reverted to the original serine residues in those positions. Additionally, the maltose bound to MalE was removed. In the pre-hydrolysis structure (PDB code: 3RLF), the reconstructed segments were the residues 371 to 374 in MalE, and residues 10 to 28, 242 to 248, 504 to 505 and 280 to 296 in MalF. The ANP-PNP molecule was replaced by ATP. Afterwards, each protein was embedded in a pre-equilibrated 480 POPC membrane. The optimal protein orientation was determined using LAMBADA^43^ and the oriented protein was inserted in the membrane manually, removing lipid molecules that were within a 1.2 Å distance cut-off.

In order to obtain the protonation states taking into account the effect of membrane environment, first we build a temporary version of the system. The protein was inserted in the membrane as described in the above paragraph. The system was further solvated and neutralized. This system was minimized in two stages, using a maximum of 50,000 steps each: the first, using position restraints of 1000 kJ/mol in all the heavy atoms, and the second stage with position restraints of the same magnitude in the protein–ligand complex heavy atoms only. An equilibration step was necessary to allow optimal membrane fitting to the protein. The equilibration phase was also performed in two stages: the first contemplated the usage of position restraints of 1000 kJ/mol in the protein–ligand complex heavy atoms, at constant temperature and pressure during 500 ps. The Berendsen baths were used for controlling temperature and pressure, with coupling constants of 0.1 and 1 ps respectively. The second stage equilibration was done using position restraints of 10,000 kJ/mol in the complex heavy atoms for 20 ns in the same conditions.

Finally, the protonation states of the protein were determined at pH 7.0 using the PETIT and MEAD packages^44,45^ and a final system was built using this information and the above described protocol for membrane insertion. The final equilibration protocol is described in Sect. 2.2. The detailed protonation states of the groups are described in tables S1 and S2.

### Simulation setup

The equilibrium MD simulations were performed using GROMACS 5.0.7^46^, along with the GROMOS 54A7 force field. The POPC parameters used were the ones derived by Poger at al^47^ and the GROMOS53A6 CARBO parameters were used to model maltose^48^. The final protonation states of ATP and ADP were the same used in Oliveira et al.^23,49,50^ and Damas et al.^51^ corresponding to a charge of − 4, − 3, respectively. A charge of −1 was assigned to the phosphate ion. The integration time step used was 2 fs. The systems were simulated using periodic boundary conditions and at constant temperature and pressure. Ions were added in order to neutralize the system. The temperature was set to 303 K using a velocity-rescale heat bath^52^, with a coupling constant of 0.1 ps with two coupling groups: one for the protein-nucleotides complex and another for the solvent and ions. The pressure was kept around 1 atm by semi-isotropic coupling with a Parrinello-Rahman bath with a coupling constant of 2.0 ps and a compressibility of 4.6 × 10^−5^ bar^−1^^53^. A cut-off of 1.0 nm was used in the calculation of van der Waals interactions. Long range electrostatic interactions were treated using the particle mesh Ewald (PME)^54,55^ method using a real-space cut-off of 1.0 nm. All neighbour lists were updated every 10 steps. All bonds were constrained to their equilibrium lengths using the LINCS^56^ algorithm, except for the water molecules in which the SETTLE algorithm was used to constrain its bonds^57^. The SPC model for water was used^58^. After system building, its potential energy was minimized in three stages with a maximum of 50,000 steps each: the first using position restraints of 1000 kJ/mol/nm on all the heavy atoms of the system, the second with position restraints of the same magnitude, but on the complex heavy atoms only, and the third with position restraints on the C-α atoms only. The equilibration stage was also done in three steps: the first one for 1 ns with position restraints of 1000 kJ/mol/nm on all the heavy atoms of the system, the second one for 1 ns with position restraints on the heavy atoms of the protein and the last one with position restraints on the C-α atoms.

Nine replicates of the pre hydrolysis states were simulated for 300 ns, while the post hydrolysis state was simulated for 360 ns and the apo state for 410 ns. The first 40 ns of the pre hydrolysis state were discarded as equilibration time, as well as the first 100 ns for the post hydrolysis state and the first 150 ns of the apo state. The effective simulation time analysed was 260 ns.

### Generation of the post-hydrolysis state

The post-hydrolysis state was generated extracting conformations from the ATP state at 20 ns. This timeframe was chosen to maximize the probability of collecting catalytically competent conformations, prior to the generation of the post-hydrolysis state. We have defined the following criteria to assess if a conformation is catalytically competent: the catalytic residues H192 and E159 must be oriented towards ATP, with a water molecule coordinated by both residues. Additionally, in order to ensure contact between ATP and the active site motifs, only conformation with an intermotif (ABC-Walker A) distance lower than 1.2 nm were considered.

Similarly to previous works^23,49,59,60^, the post hydrolysis state was generated using the slow-growth method, making the transformation from ATP to ADP and phosphate coupled to a lambda parameter that varies from 0 to 1, allowing a smooth conversion of the molecules. In this case we are not interested in calculating the free energy associated with this transformation. Nonetheless, in the present case, the entire hydrolysis process was divided in two stages. The first step mimics the hydrolysis process, while the second mimics the catalytic residue regeneration. In the first step the conversion of ATP to ADP and phosphate is simulated, along with the annihilation of a proton in H192 and the creation of a proton in E159. In this way, the final state agrees with recently proposed catalytic mechanisms for ATP hydrolysis in the maltose importer^21^. In the second stage of hydrolysis there is the regeneration of the proton states of residues H192 and E159. The first step, that simulated the hydrolysis process was performed for 5 ps using a timestep of 0.0005 ps, while the regeneration step was done for 2 ps, using the same timestep. After both slow-growth procedures, an extra positive charge is added to neutralize the system, in the form of a sodium ion replacing a water molecule far away from the protein. This allows to avoid inconsistencies due to the PME algorithm. These simulations were performed using the same conditions as described above.

### Analysis of equilibrium MD simulations

Analyses were done using the tools included in the GROMACS 5.0.7 package.

The harmonic ensemble similarity (D_HES_) is a measure of the similarity between ensembles and was derived by Lindorff-Larsen et al.^61^. We have used the implementation available in the ENCORE toolkit^62^ included in the MDAnalysis package^63,64^. D_HES_ was calculated between the ADP and ATP states using the C-α positions of each individual residue. The trajectories were previously fitted to a common structure and the comparisons were made in a cross-replicate way, i.e., each replicate was compared to all other replicates, apart from itself. The displayed values correspond to the average for all comparisons for each residue. The pore radius was measured using HOLE, assigning the GROMOS atomic radii^65^. The secondary structure was determined using the DSSP program. The elements of secondary structure considered were α-helix, β-sheet, β-bridge, turn, and 3_10_ helix, according to the DSSP classification^66^.

Principal components analysis (PCA) was performed using the atomic coordinates of the C-α atoms, obtained during the equilibrium MD simulations. We followed the protocol defined in Campos et al.^67^. First, a central structure of the trajectory set is determined, being this structure the one that minimizes the total dispersion of the average of the squared rmsd. This central structure is then used for fitting the whole trajectory used for the PCA calculation, and as reference structure for the calculation of the covariance matrix. Afterwards, PCA of the covariance matrix is performed. The density maps were obtained using a two-dimensional grid with a spacing of 0.2 Å and a gaussian kernel estimator^67^.

The diffusion maps of maltose were obtained by first extracting the center-of-mass coordinates using MDAnalysis^63,64^ and the probability densities were estimated via a three-dimensional grid with a spacing of 0.05 nm^3^ using a gaussian kernel estimator^67^.

The 3D structure maps were obtained using PDBsum server^68^ and the D_HES_ were mapped with the aid of PyMOL 2.0.

All the error bars presented in the histograms correspond to the 95% confidence interval obtained by bootstrapping^69^.

PyMOL 2.0 was used to visualize trajectories and produce the pictures presented^70^. The plots were produced using the Matplotlib^71^ package in Python and Gnuplot^72^.

### Potential of mean force (PMF) calculations

The PMF profiles were calculated using the umbrella sampling method. Pulling simulations were performed with the goal of generating initial conformations for umbrella sampling windows. GROMACS 2018.4^73^ was used for pulling and umbrella sampling simulations, in the same conditions as the equilibrium MD, but this time setting an isotropic pressure coupling, to avoid fluctuations of the box in the zz’ dimension that affect the reaction coordinate, which is also in the zz’ direction. Pulling simulations were started from frames extracted from equilibrium MD simulations at several times. In these frames, the maltose molecules were at random positions along the pore as a result of their diffusion. In order to span the entire pore length, bidirectional pulling was made. In the direction towards the NBDs, the reaction coordinate used was the z-component of the distance between the center of mass of maltose and V442. The simulations were stopped as maltose reached the level of the coupling helices. An harmonic potential was applied to this reaction coordinate, with a force constant of 1000 kJ/mol/nm^2^ and a constant velocity of 3.5 Å/ns was used in the pre and post hydrolysis states, while 2.5 Å/ns was used in the nucleotide-free state. The systems were simulated for a maximum of 50 ns. In the direction towards MalE, the reaction coordinate used was the z-component of the distance between the center of mass of maltose and MalE. In a similar way to the previous simulations, a harmonic potential was applied to this reaction coordinate, with a force constant of 1000 kJ/mol/nm^2^. The conformations extracted from equilibrium MD simulations were simulated for a maximum of 20 ns. In the ATP simulation, the pulling velocity used was of 2.5 Å/ns. In the ADP simulation, 20 ns were simulated at the pulling velocity of 1.5 Å/ns, along with more 13 ns at the pulling velocity of 3.5 Å/ns. In the Apo state, 20 ns were simulated at the pulling velocity of 2.5 Å/ns, along with an extra 16 ns at the pulling velocity of 3.5 Å/ns. The extra step with increased velocity was necessary to overcome the steric hindrance caused by the different conformation of the transmembrane domains and reaching the periplasmic gate level.

For the umbrella sampling simulations, the PLUMED 2.5.1 plugin was used. The windows were initially spaced by 0.06 nm, but depending on the window behaviour, further windows were necessary in certain regions, while in other regions, windows were removed to avoid excessive overlap. The final list of windows used for each PMF can be found in supplementary information in tables S3 to S6. Harmonic restraints were used with a force constant of 500 kJ/mol/nm^2^ or 800 kJ/mol/nm^2^, depending on the difficulty of sampling the region. The restraint potential was applied to the z-component of the center of mass of maltose. The umbrella windows were simulated for 50 ns, in which the first 20 ns were discarded as the equilibration period. The PMF profiles were obtained using the Umbrella Integration method by Kästner et al.^74,75^. We used the code created by Stroët et al.^76^. The orientation angle of maltose was calculated using MDAnalysis^63^. This angle is defined as the angle between the z-axis and the vector that links the most distant carbon atoms in maltose rings.

The probability densities maps for the analysing umbrella sampling simulations were obtained by estimating densities in a two-dimensional grid with a spacing of 0.1 hydrogen bonds/nm, 0.1 deg/nm and 0.1 Å/nm using a Gaussian Kernel estimator^67^.

## Supplementary Information

Supplementary Information.
