# Supplementary material for: ATP hydrolysis and nucleotide exit enhance maltose translocation in the MalFGK2E importer
Source: Sci Rep. 2021 May 19;11:10591. doi: 10.1038/s41598-021-89556-y (PMC8134467; doi:10.1038/s41598-021-89556-y)
Supplement: Supplementary file 1 — Supplementary Information. [file 41598_2021_89556_MOESM1_ESM.pdf]

Supplementary Information

ATP hydrolysis and nucleotide exit enhance maltose translocation in the MalFGK$_2$E importer

Bárbara Abreu[1], Carlos Cruz[1], A. Sofia F. Oliveira[1,2], Cláudio M. Soares[1]

1-ITQB NOVA, Instituto de Tecnologia Química e Biológica António Xavier, Universidade Nova de Lisboa, Oeiras, Portugal

2-School of Biochemistry & Centre for Computational Chemistry, University of Bristol, Bristol, UK

Table S1-Protonation states of the histidine residues for the ADP and ATP states.

	**MalE**	
**Residue**	**Protonation**	**Charge**
**H39**	Protonated in both Nδ and Nε	+1
**H64**	Protonated in Nε	0
**H203**	Protonated in Nδ	0
	**MalG**	
**Residue**	**Protonation**	**Charge**
**H17**	Protonated in Nε	0
**H58**	Protonated in both Nδ and Nε	+1
**H159**	Protonated in Nε	0
**H173**	Protonated in Nε	0
	**MalK**	
**Residue**	**Protonation**	**Charge**
**H27**	Protonated in both Nδ and Nε	+1
**H89**	Protonated in Nε	0
**H125**	Protonated in Nε	0
**H180**	Protonated in Nε	0
**H192**	Protonated in both Nδ and Nε	+1
**H223**	Protonated in Nε	0
**H289**	Protonated in Nε	0
**H317**	Protonated in Nε	0
**H353**	Protonated in Nε	0
**H366**	Protonated in both Nδ and Nε	+1

Table S2- Protonation states of the histidine residues for the Apo state.

	**MalE**	
**Residue**	**Protonation**	**Charge**
**H39**	Protonated in both Nδ and Nε	+1
**H64**	Protonated in Nε	0
**H203**	Protonated in Nδ	0
	**MalG**	
**Residue**	**Protonation**	**Charge**
**H17**	Protonated in Nε	0
**H58**	Protonated in both Nδ and Nε	+1
**H159**	Protonated in Nε	0
**H173**	Protonated in Nε	0

MalK		
**Residue**	**Protonation**	**Charge**
**H27**	Protonated in both Nδ and Nε	+1
**H89**	Protonated in Nε	0
**H125**	Protonated in Nε	0
**H180**	Protonated in Nε	0
**H192**	Protonated in Nε	0
**H223**	Protonated in Nε	0
**H289**	Protonated in Nε	0
**H317**	Protonated in Nε	0
**H353**	Protonated in Nε	0
**H366**	Protonated in both Nδ and Nε	+1

Conformational drift of the equilibrium MD simulations

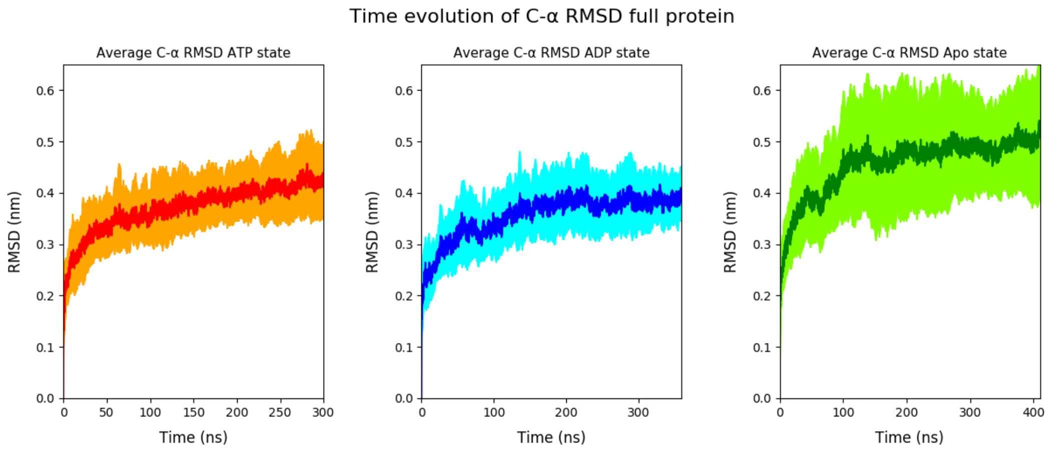

Figure S1- Temporal evolution of the average C-α RMSD for full $MalFGK_2E$ complex, in the three simulated states: the ATP (pre-hydrolysis) state, the ADP (post-hydrolysis) state and the Apo (nucleotide-free) state. Each state comprises nine replicates. The C-α RMSD was calculated against the initial structure of each simulation. The error bars correspond to the standard deviation obtained by bootstrapping. The error bars are represented in orange, cyan and light green in the ATP, ADP and Apo states respectively. The length of the simulations is different in the three states, to ensure equilibrated simulations: the ATP state was simulated for 300 ns, the ADP for 360 ns and the Apo for 410 ns.

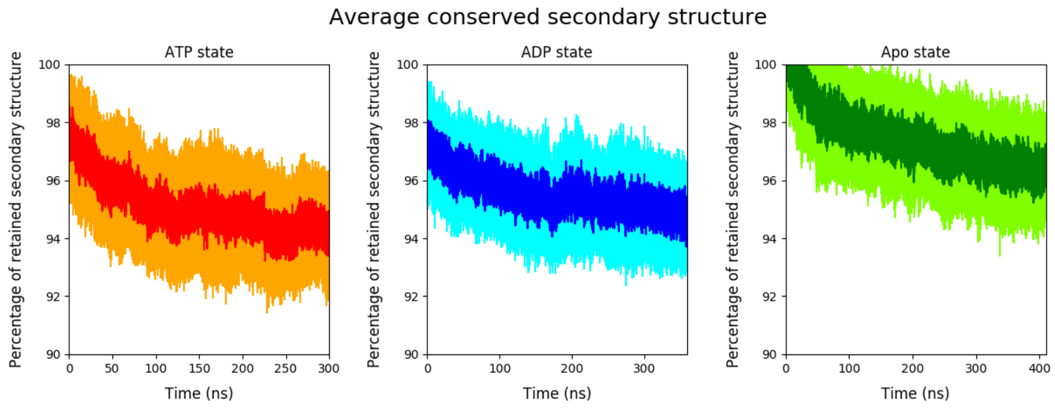

Figure S2- Percentage of retained secondary structure for all the simulated states. Each state comprises nine replicates. The error bars correspond to the standard deviation obtained by bootstrapping. The error bars are represented in orange, cyan and light green in the ATP, ADP and Apo states respectively. The length of the simulations is different in the three states, to ensure equilibrated simulations: the ATP state was simulated for 300 ns, the ADP for 360 ns and the Apo for 410 ns.

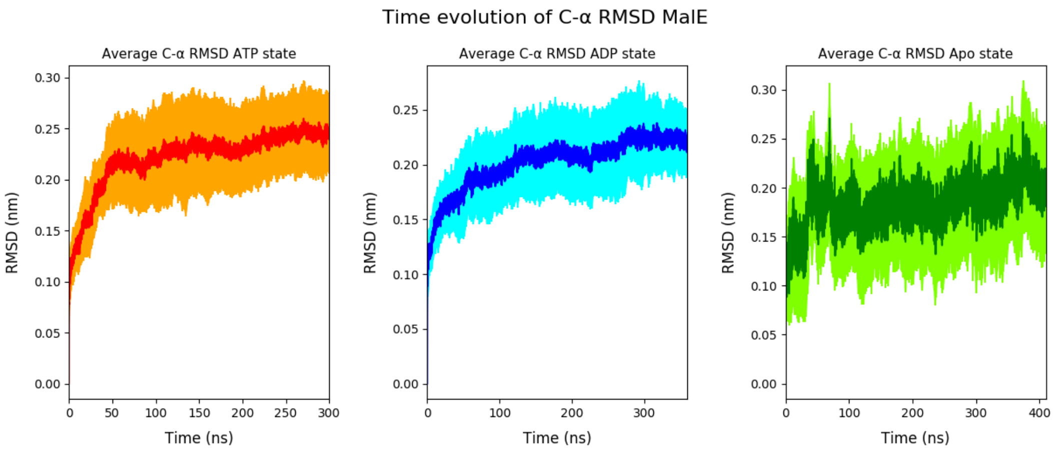

Figure S3- Temporal evolution of the average C-α RMSD for MalE, in the three simulated states: the ATP (pre-hydrolysis) state, the ADP (post-hydrolysis) state and the Apo (nucleotide-free) state. Each state comprises nine replicates. The C- α RMSD was calculated against the initial structure of each simulation. The error bars correspond to the standard deviation obtained by bootstrapping. The error bars are represented in orange, cyan and light green in the ATP, ADP and Apo states respectively. The length of the simulations is different in the three states, to ensure equilibrated simulations: the ATP state was simulated for 300 ns, the ADP for 360 ns and the Apo for 410 ns.

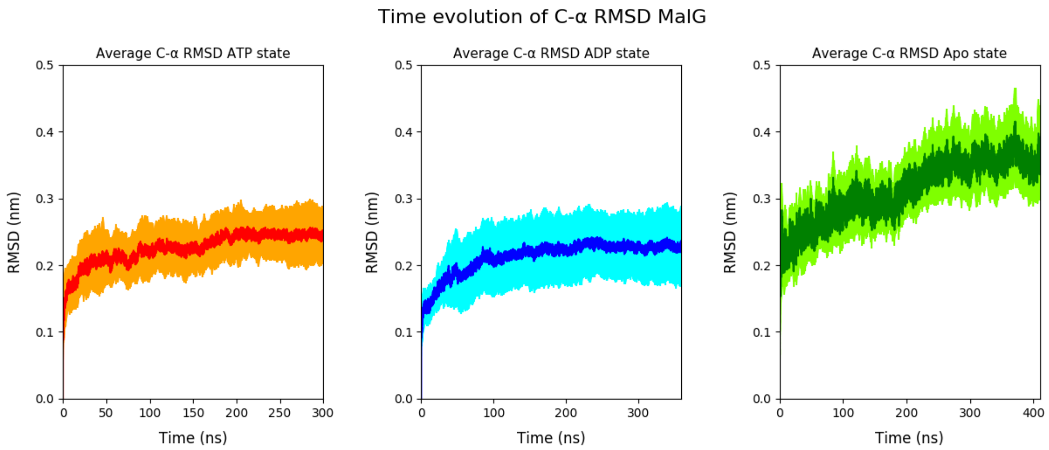

Figure S4- Temporal evolution of the average C-α RMSD for MalG, in the three simulated states: the ATP (pre-hydrolysis) state, the ADP (post-hydrolysis) state and the Apo (nucleotide-free) state. Each state comprises nine replicates. The C-α RMSD was calculated against the initial structure of each simulation. The error bars correspond to the standard deviation obtained by bootstrapping. The error bars are represented in orange, cyan and light green in the ATP, ADP and Apo states respectively. The length of the simulations is different in the three states, to ensure equilibrated simulations: the ATP state was simulated for 300 ns, the ADP for 360 ns and the Apo for 410 ns.

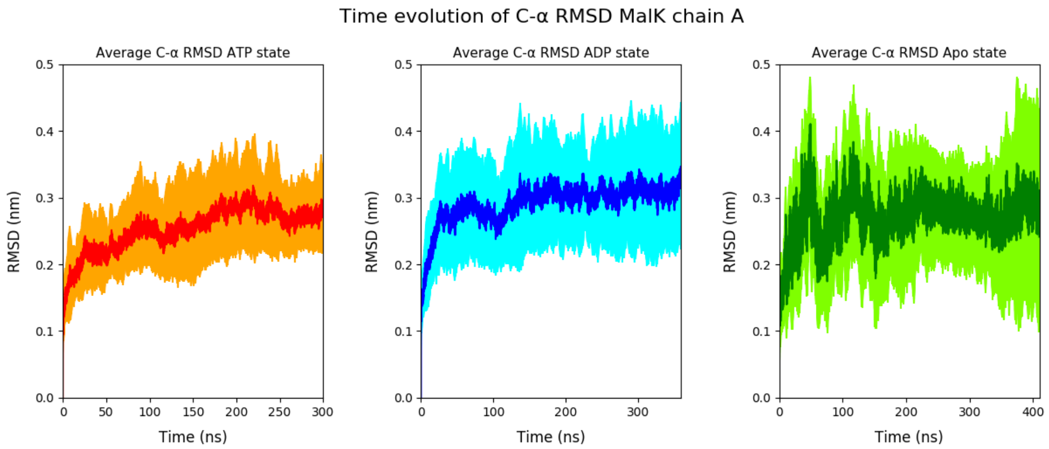

Figure S5- Temporal evolution of the average C-α RMSD for MalK chain A, in the three simulated states: the ATP (pre-hydrolysis) state, the ADP (post-hydrolysis) state and the Apo (nucleotide-free) state. Each state comprises nine replicates. The C-α RMSD was calculated against the initial structure of each simulation. The error bars correspond to the standard deviation obtained by bootstrapping. The error bars are represented in orange, cyan and light green in the ATP, ADP and Apo states respectively. The length of the simulations is different in the three states, to ensure equilibrated simulations: the ATP state was simulated for 300 ns, the ADP for 360 ns and the Apo for 410 ns.

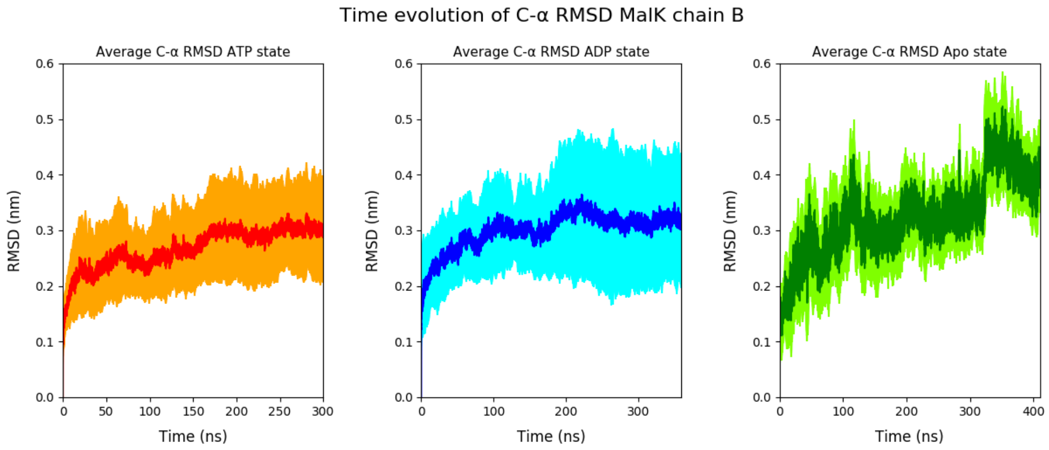

Figure S6- Temporal evolution of the average C-α RMSD for MalK chain B, in the three simulated states: the ATP (pre-hydrolysis) state, the ADP (post-hydrolysis) state and the Apo (nucleotide-free) state. Each state comprises nine replicates. The C-α RMSD was calculated against the initial structure of each simulation. The error bars correspond to the standard deviation obtained by bootstrapping. The error bars are represented in orange, cyan and light green in the ATP, ADP and Apo states respectively. The length of the simulations is different in the three states, to ensure equilibrated simulations: the ATP state was simulated for 300 ns, the ADP for 360 ns and the Apo for 410 ns.

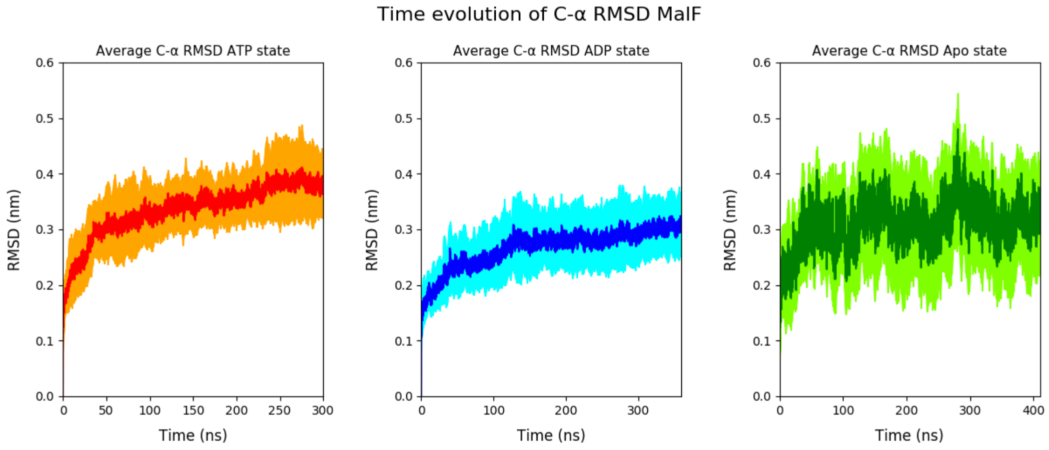

Figure S7- Temporal evolution of the average C-α RMSD for MalF, in the three simulated states: the ATP (pre-hydrolysis) state, the ADP (post-hydrolysis) state and the Apo (nucleotide-free) state. Each state comprises nine replicates. The C-α RMSD was calculated against the initial structure of each simulation. The error bars correspond to the standard deviation obtained by bootstrapping. The error bars are represented in orange, cyan and light green in the ATP, ADP and Apo states respectively. The length of the simulations is different in the three states, to ensure equilibrated simulations: the ATP state was simulated for 300 ns, the ADP for 360 ns and the Apo for 410 ns.

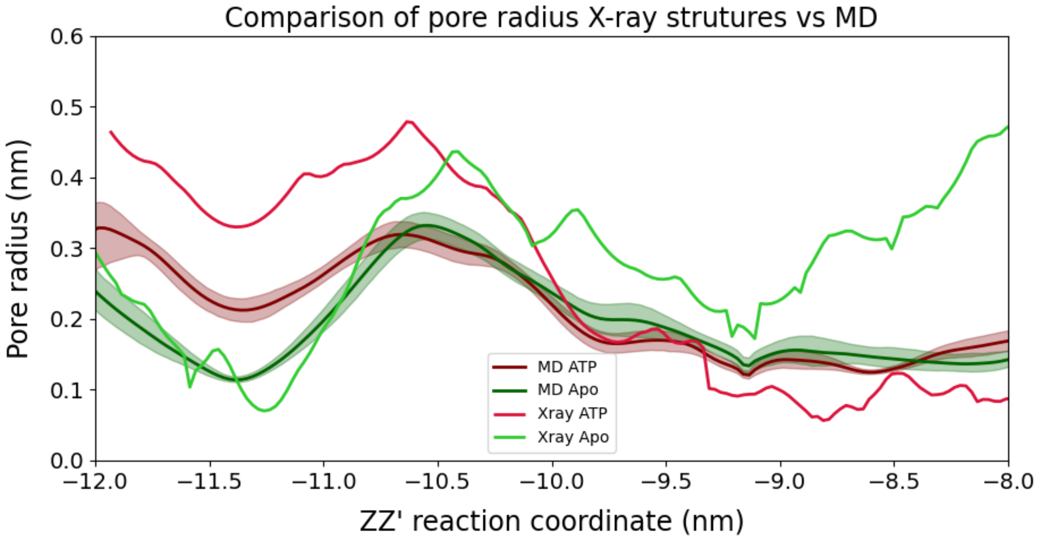

Figure S8- Comparison of the pore radius between the X-ray structures used as starting points for this work and the MD simulations of the respective states, ATP and Apo. The radius of the starting structure of the ATP state is represented in red, while the radius of the x-ray structure for the Apo state is represented in green. The average radius from the ATP MD simulation is presented in dark red, while average radius from the Apo MD simulation is presented in dark green. The error shading is the 95% confidence interval obtained by bootstrapping.

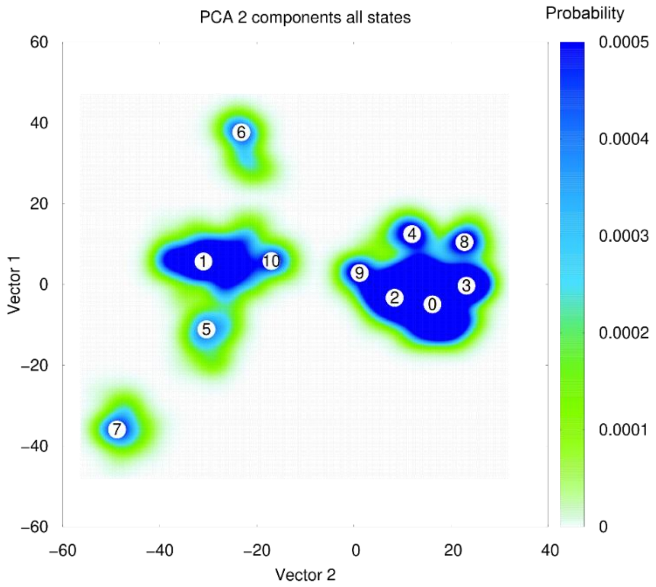

Figure S9- Probability landscape obtained when performing PCA of all the simulated states (ATP, ADP and Apo). The probability is plotted in a gradient of colour from white (lower probability) to dark blue (higher probability). The white dots with numbers indicate the maxima of each population. The basins 1, 5, 6, 7 and 10 are exclusively occupied by the Apo state, while basins 0, 2, 3, 4, 8 and 9 are occupied by the ATP and ADP states.

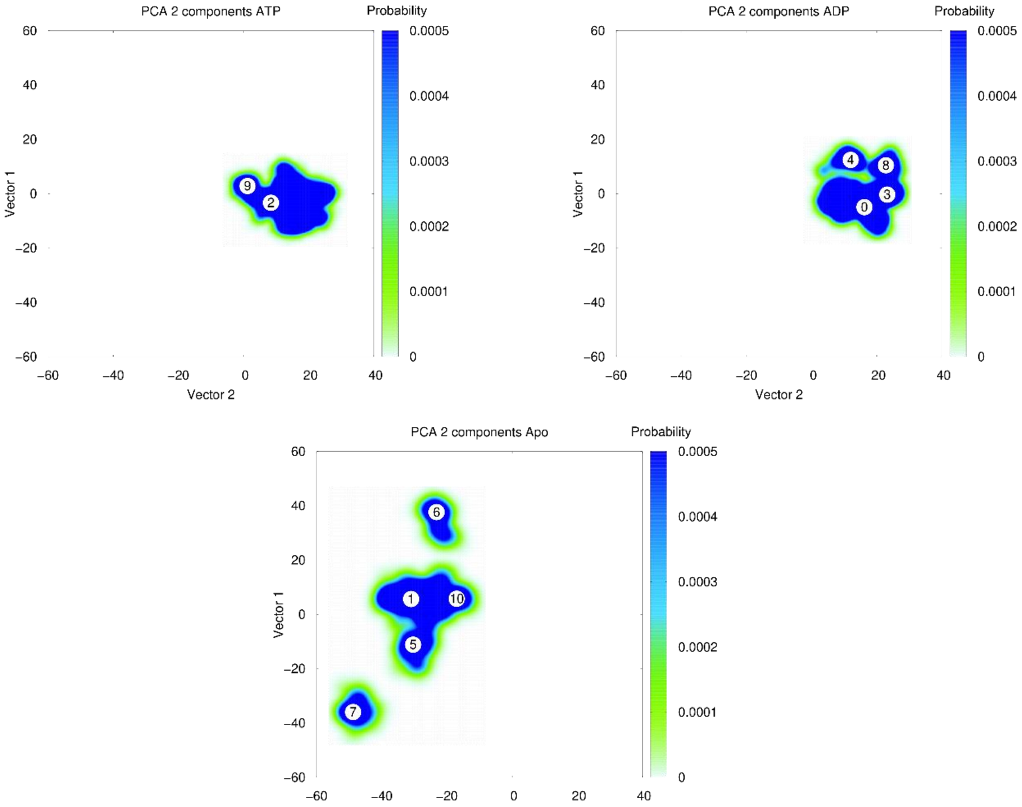

Figure S10- Probability landscape of each simulated state, projected in the same space as the landscape in figure S9. The probability is plotted in a gradient of colour from white (lower probability) to dark blue (higher probability). The white dots with numbers indicate the maxima of each basin.

Table S3-Distribution of the conformations of three states: ATP, ADP and Apo per region. The lines coloured in green correspond to regions predominantly Apo, the lines coloured in blue correspond to regions with a predominant ADP character and the line in red corresponds to a region with a high ATP content.

Basin	%ATP	%ADP	%Apo
**0**	66.84	45.33	0.00
**1**	0.00	0.00	59.11
**2**	15.42	18.56	0.00
**3**	9.16	14.85	0.00
**4**	1.43	10.43	0.00
**5**	0.00	0.00	11.55
**6**	0.00	0.00	11.11
**7**	0.00	0.00	11.11
**8**	0.07	10.23	0.00
**9**	7.09	0.61	0.00
**10**	0.00	0.00	7.12

Figure S11

A

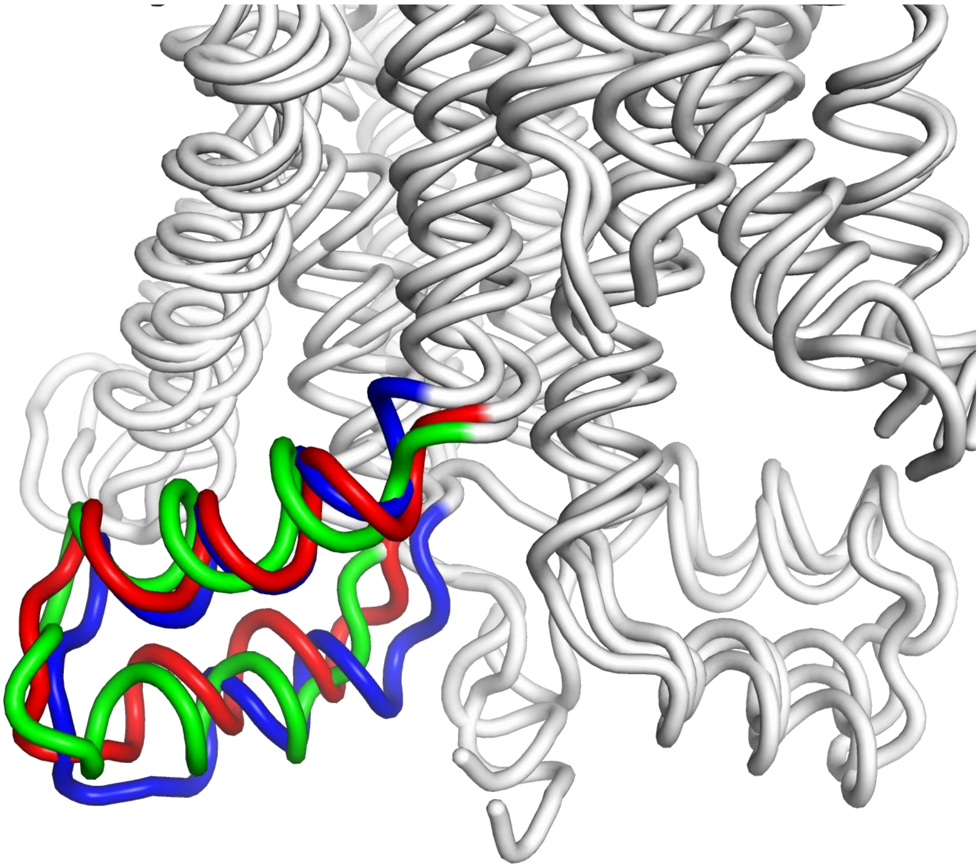

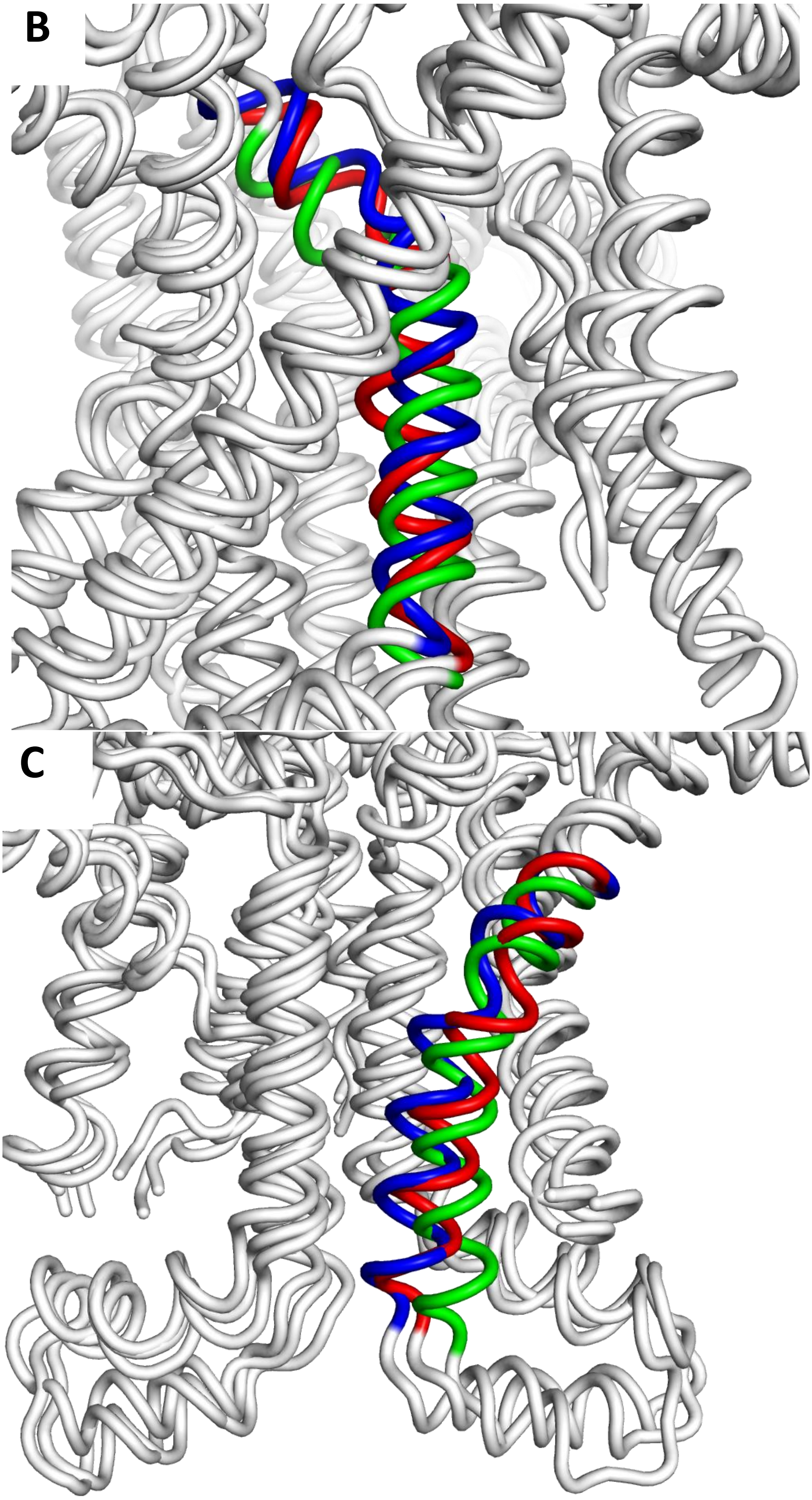
B
C

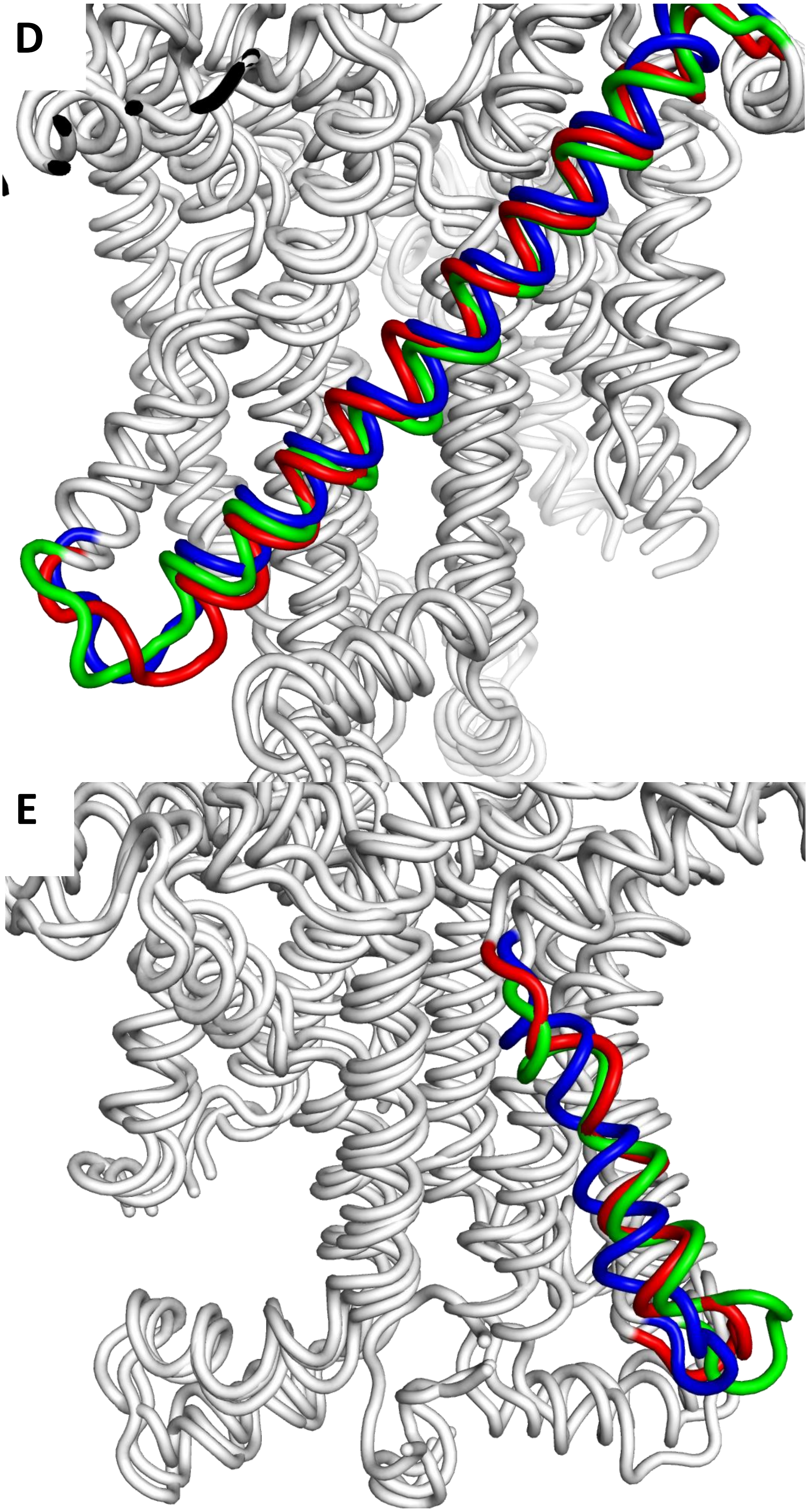
D
E

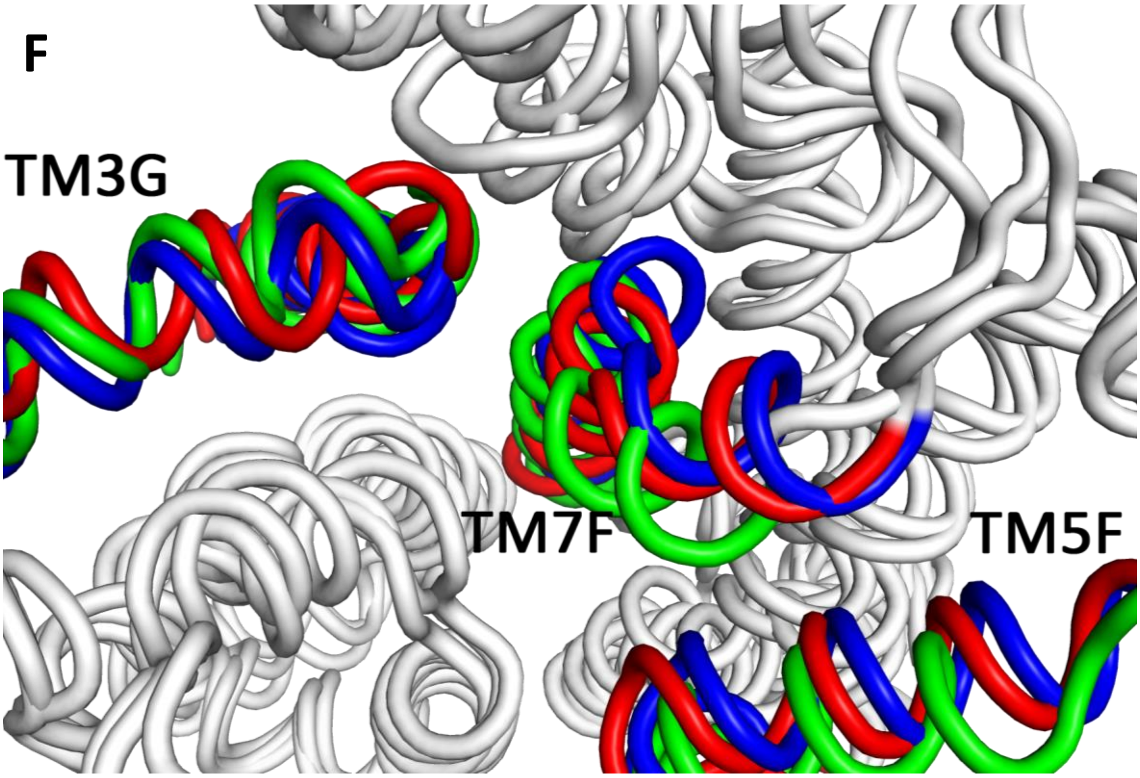

Figure S11- Comparison of the ATP, ADP and Apo most probable structures. The conformations represented are the most probable structures of relevant basins, namely basins 8, 9 and 2, that correspond to the ADP, ATP and Apo states, respectively. The most relevant regions are coloured while the remaining protein is represented in white. The most probable ATP conformation is represented in red, while its ADP counterpart is represented in blue and the Apo conformation is represented in green. The orientation of the figures A to E is from the periplasmic side to the cytoplasmic one, while figure F is focusing the periplasmic side from the bottom. A - The MalF coupling helix. B- Transmembrane helix 7 of MalF (TM7F). C- Transmembrane helix 6 of MalF (TM6F). D- Transmembrane helix 4 of MalF (TM4F). E- Transmembrane helix 5 of MalF (TM5F). F- Bottom view of the pore highlighting the TM3G, TM7F and TM5F helices.

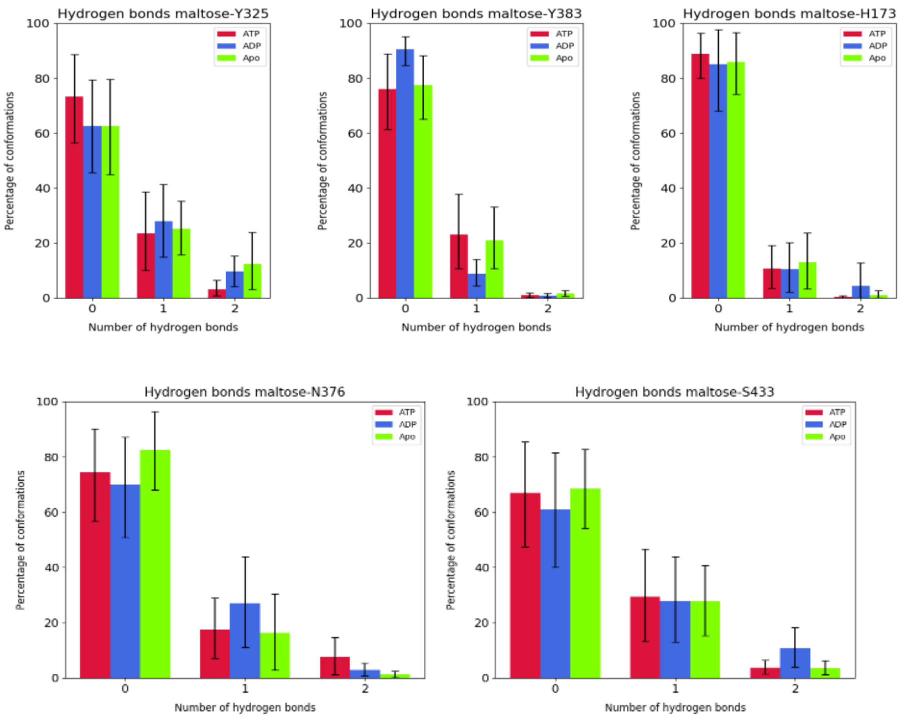

Figure S12-Number of hydrogen bonds made between maltose and key protein residues Y325, Y383, H173, N376 and S433. ATP is represented in red, ADP in blue and the Apo state in green. The error bars represent the 95% confidence interval obtained by bootstrapping.

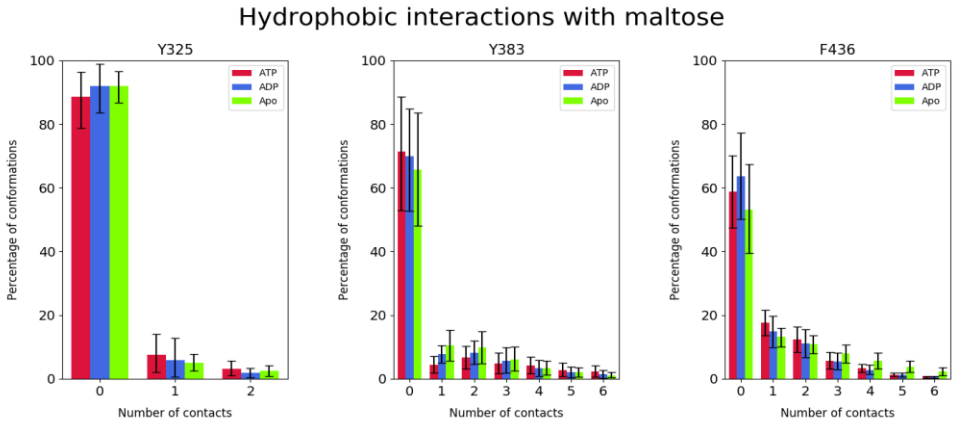

Figure S13-Number of hydrophobic contacts made between the maltose rings and the aromatic sidechains of residues Y325, Y383 and F436. The maximum distance considered for a contact is 0.4 nm. ATP is represented in red, ADP in blue and the Apo state in green. The error bars represent the 95% confidence interval obtained by bootstrapping.

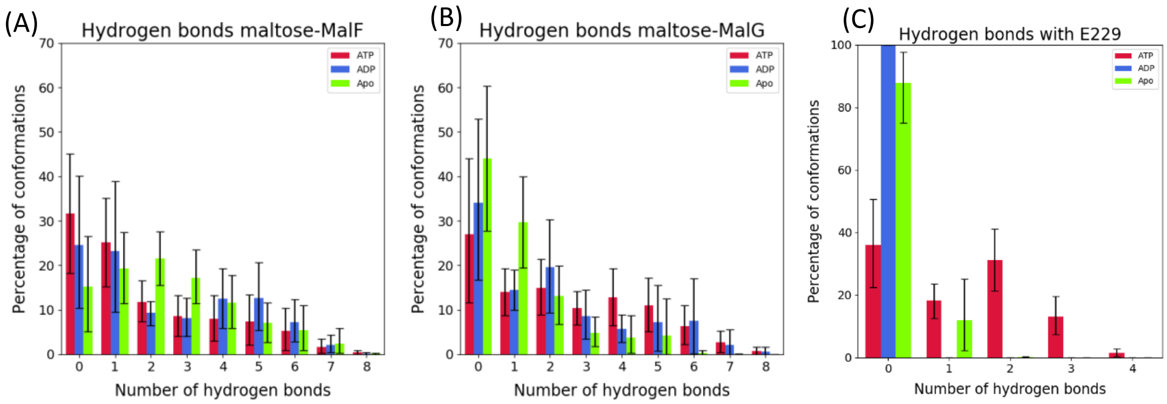

Figure S14-Number of hydrogen bonds made between maltose and protein residues. (A)-Hydrogen bonds between maltose and MalF. (B)- Hydrogen bonds between maltose and MalG. (C)-Hydrogen bonds between maltose and E229. ATP is represented in red, ADP in blue and the Apo state in green. The error bars represent the 95% confidence interval obtained by bootstrapping.

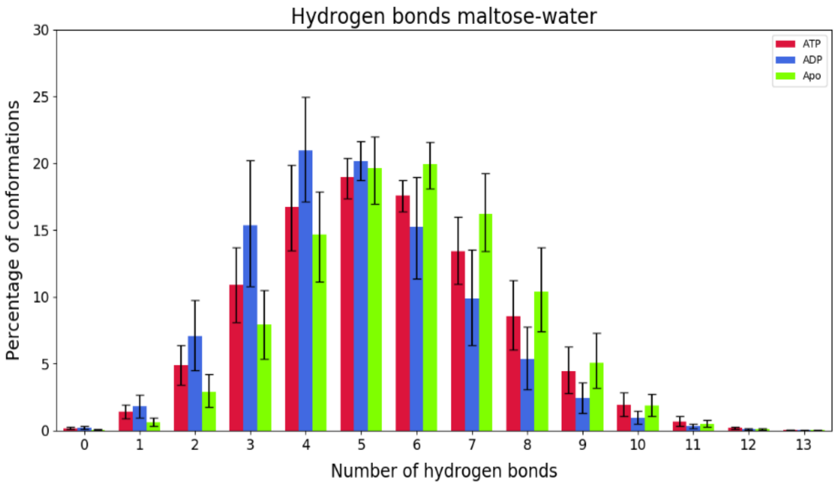

Figure S15-Number of hydrogen bonds made between maltose and water molecules within the pore. ATP is represented in red, ADP in blue and the Apo state in green. The error bars represent the 95% confidence interval obtained by bootstrapping.

Table S4- Windows used for umbrella sampling of the ATP PMF profile

Initial position (nm)	Force constant ($kJ/mol/nm^2$)	Initial position (nm)	Force constant ($kJ/mol/nm^2$)
**-12.1520**	500	**-10.8662**	500
**-11.0637**	500	**-10.9405**	500
**-11.2453**	500	**-9.09825**	500
**-11.4750**	500	**-9.14214**	800
**-11.5311**	500	**-9.31624**	800
**-11.8445**	500	**-9.42136**	500
**-11.9482**	500	**-9.65039**	500
**-10.0596**	500	**-9.77419**	500
**-10.1562**	500	**-9.85904**	500
**-10.2517**	500	**-8.35064**	500
**-10.4421**	500	**-8.43524**	500
**-10.5236**	500	**-8.65938**	500
**-10.7213**	500	**-8.81653**	500

Table S5- Windows used for sampling of the ADP PMF profile, in the ADP state

Initial position (nm)	Force constant ($kJ/mol/nm^2$)	Initial position (nm)	Force constant ($kJ/mol/nm^2$)
**-12.5292**	500	**-10.2123**	500
**-12.3985**	500	**-10.0026**	500
**-12.2780**	500	**-9.86401**	500
**-12.0389**	500	**-9.64683**	500
**-11.8563**	800	**-9.45277**	500
**-11.7915**	500	**-9.39911**	500
**-11.6772**	500	**-9.33203**	500
**-11.4938**	800	**-9.10921**	500
**-11.3291**	500	**-9.04344**	500
**-11.0986**	500	**-8.80757**	500
**-10.9470**	500	**-8.69352**	500
**-10.7803**	800	**-8.55649**	800
**-10.5825**	500	**-8.43859**	800
**-10.4495**	500	**-8.25795**	500
**-10.3328**	500		

Table S6- Windows used for sampling of the Apo PMF profile

Initial position (nm)	Force constant (kJ/mol/nm^2)	Initial position (nm)	Force constant (kJ/mol/nm^2)
**-12.0641**	500	**-10.5113**	500
**-12.3097**	500	**-10.7496**	500
**-12.4238**	500	**-10.9657**	500
**-11.1371**	500	**-9.12126**	500
**-11.2258**	500	**-9.20689**	500
**-11.4573**	500	**-9.31658**	500
**-11.6909**	500	**-9.55786**	500
**-11.8254**	500	**-9.72485**	500
**-11.9576**	500	**-9.79942**	500
**-10.0387**	500	**-8.6486**	500
**-10.1531**	500	**-8.83127**	500
**-10.3285**	500	**-8.95105**	500
**-10.3874**	500		

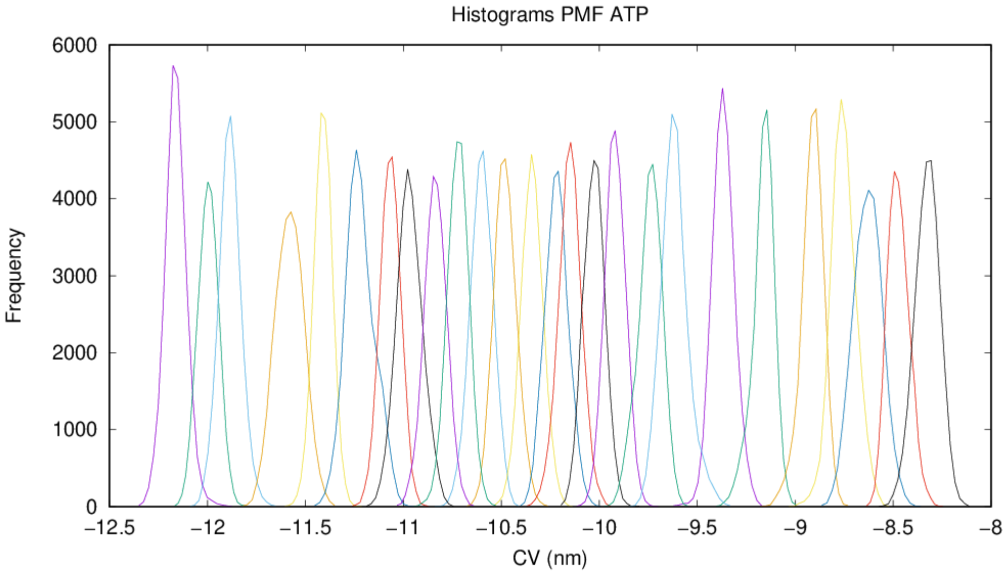

Figure S16- Umbrella histograms obtained from each window in the ATP state. Each histogram represents the reaction coordinate distribution for each window and contains 30 ns of simulation.

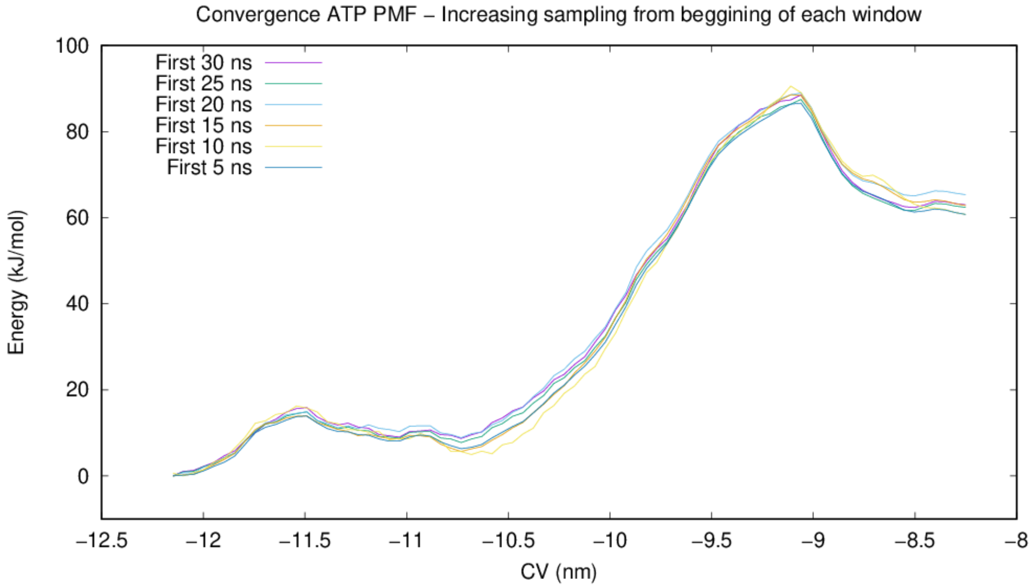

Figure S17- Evaluation of ATP PMF convergence by increasingly adding 5 ns of sampling from the beginning of each umbrella window. The PMF using the first 5 ns of each window is represented in dark blue, using the first 10 ns of each window is represented in yellow, the first 15 ns of each window is represented in orange, the first 20 ns of each window is represented in cyan, the first 25 ns of each window is represented in green and using the whole 30 ns of each window is represented in purple.

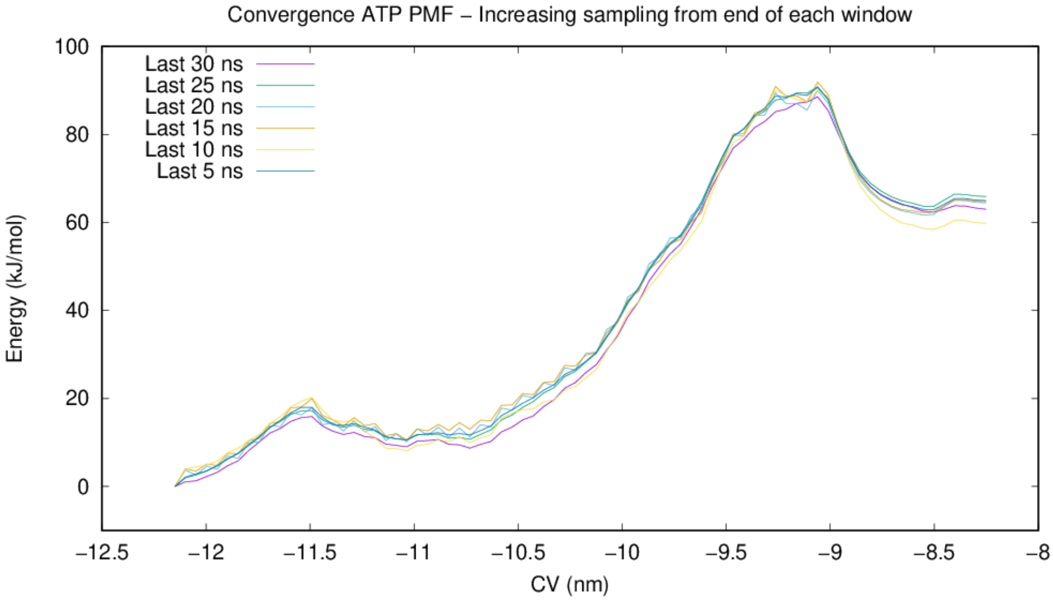

Figure S18- Evaluation of ATP PMF convergence by increasingly adding 5 ns of sampling from the end of each umbrella window. The PMF using the last 5 ns of each window is represented in dark blue, using the last 10 ns of each window is represented in yellow, the last 15 ns of each window is represented in orange, the last20 ns of each window is represented in cyan, the last 25 ns of each window is represented in green and using the whole 30 ns of each window is represented in purple.

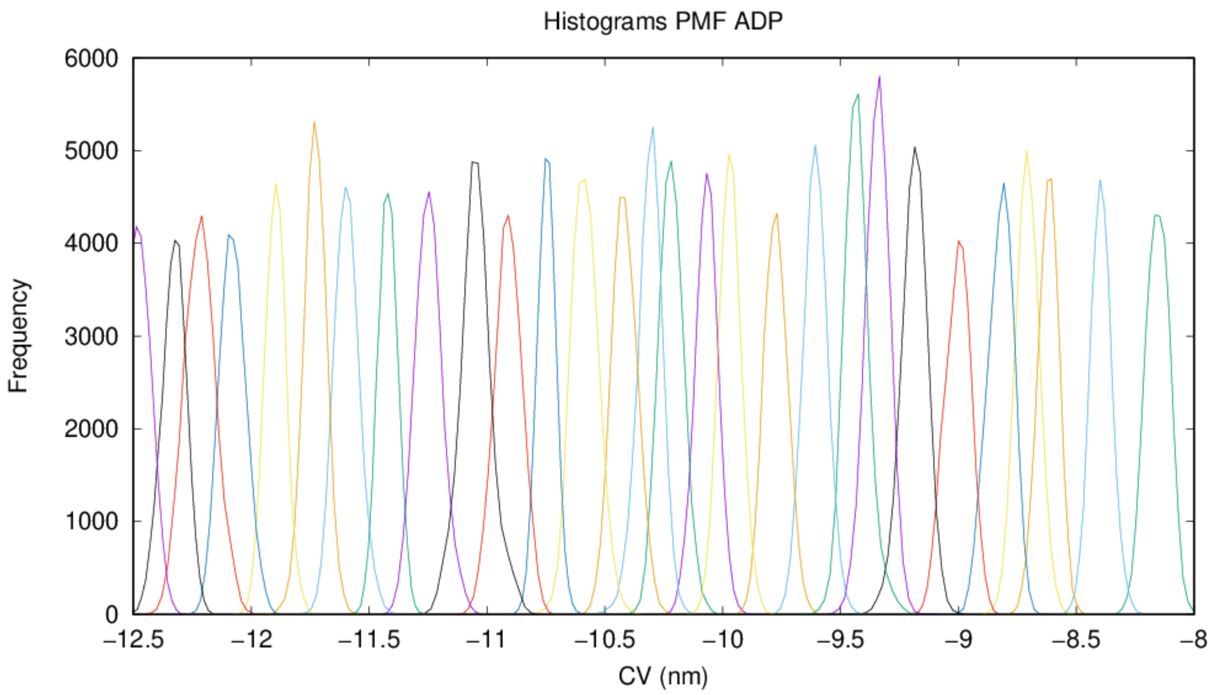

Figure S19- Umbrella histograms obtained from each window in the ADP state, for the ADP-2 PMF profile. Each histogram represents the reaction coordinate distribution for each window and contains 30 ns of simulation.

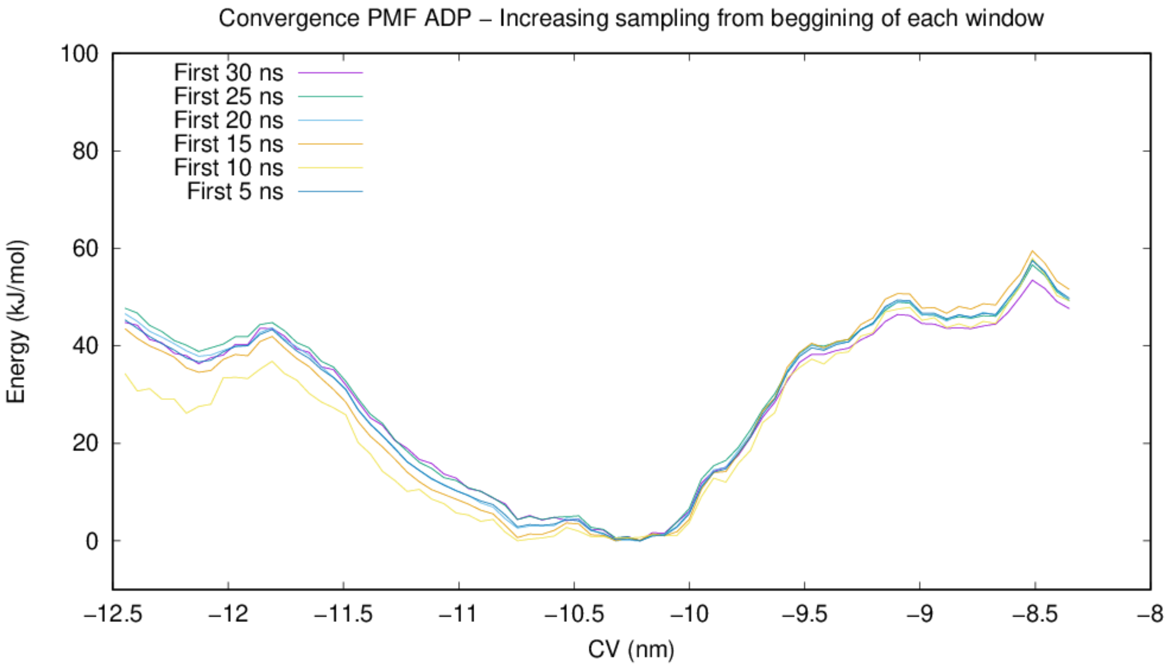

Figure S20- Evaluation of ADP PMF convergence by increasingly adding 5 ns of sampling from the beginning of each umbrella window. The PMF using the first 5 ns of each window is represented in dark blue, using the first 10 ns of each window is represented in yellow, the first 15 ns of each window is represented in orange, the first 20 ns of each window is represented in cyan, the first 25 ns of each window is represented in green and using the whole 30 ns of each window is represented in purple.

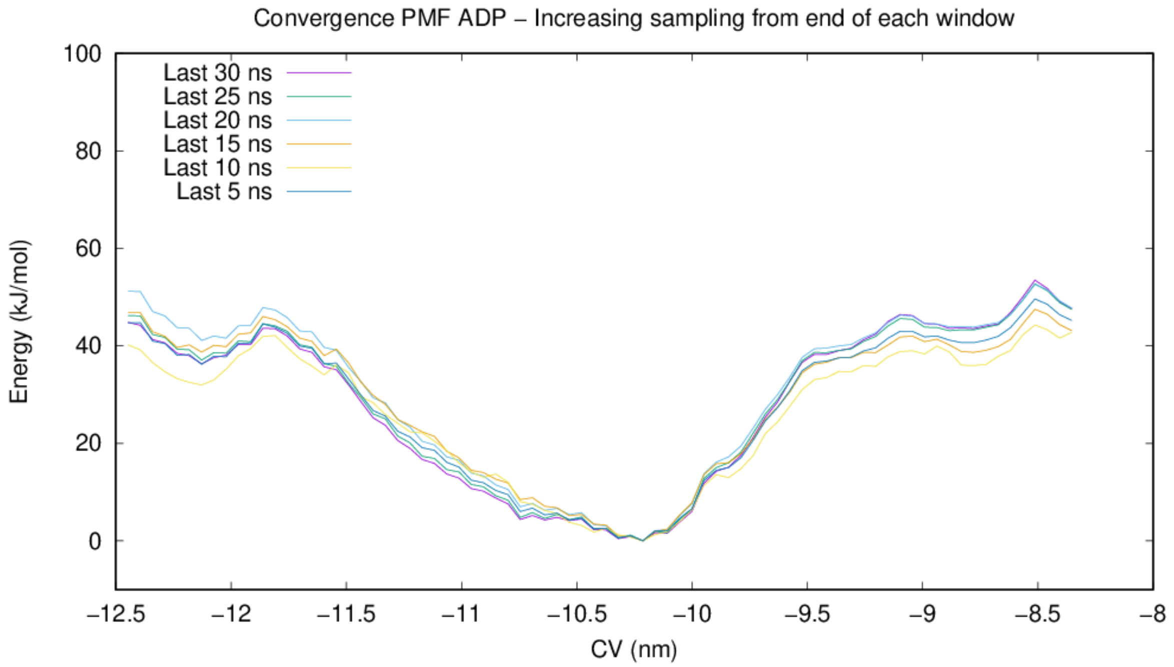

Figure S21- Evaluation of the ADP PMF convergence by increasingly adding 5 ns of sampling from the end of each umbrella window. The PMF using the last 5 ns of each window is represented in dark blue, using the last 10 ns of each window is represented in yellow, the last 15 ns of each window is represented in orange, the last20 ns of each window is represented in cyan, the last 25 ns of each window is represented in green and using the whole 30 ns of each window is represented in purple.

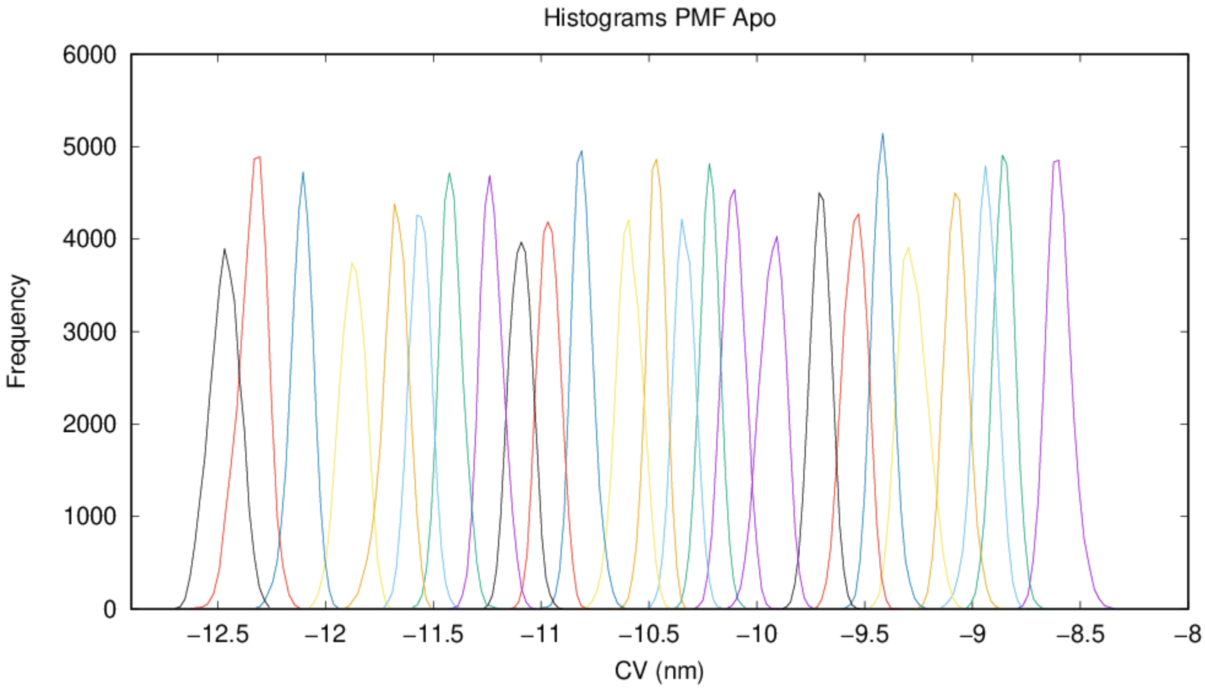

Figure S22- Umbrella histograms obtained from each window in the Apo state. Each histogram represents the reaction coordinate distribution for each window and contains 30 ns of simulation.

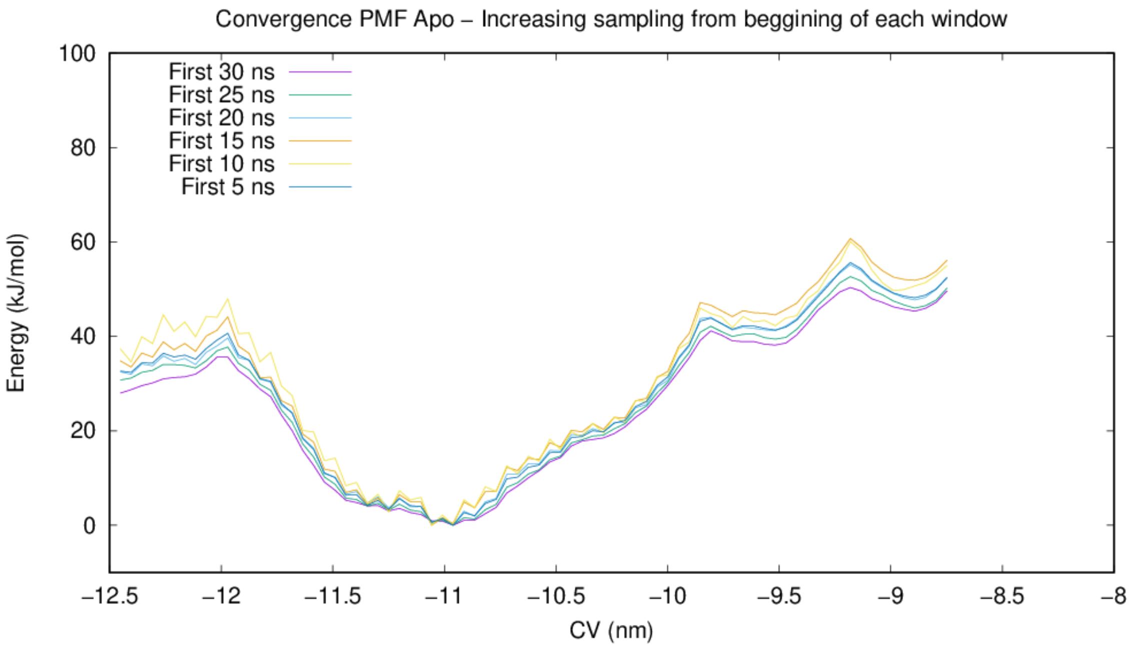

Figure S23- Evaluation of Apo PMF convergence by increasingly adding 5 ns of sampling from the beginning of each umbrella window. The PMF using the first 5 ns of each window is represented in dark blue, using the first 10 ns of each window is represented in yellow, the first 15 ns of each window is represented in orange, the first 20 ns of each window is represented in cyan, the first 25 ns of each window is represented in green and using the whole 30 ns of each window is represented in purple.

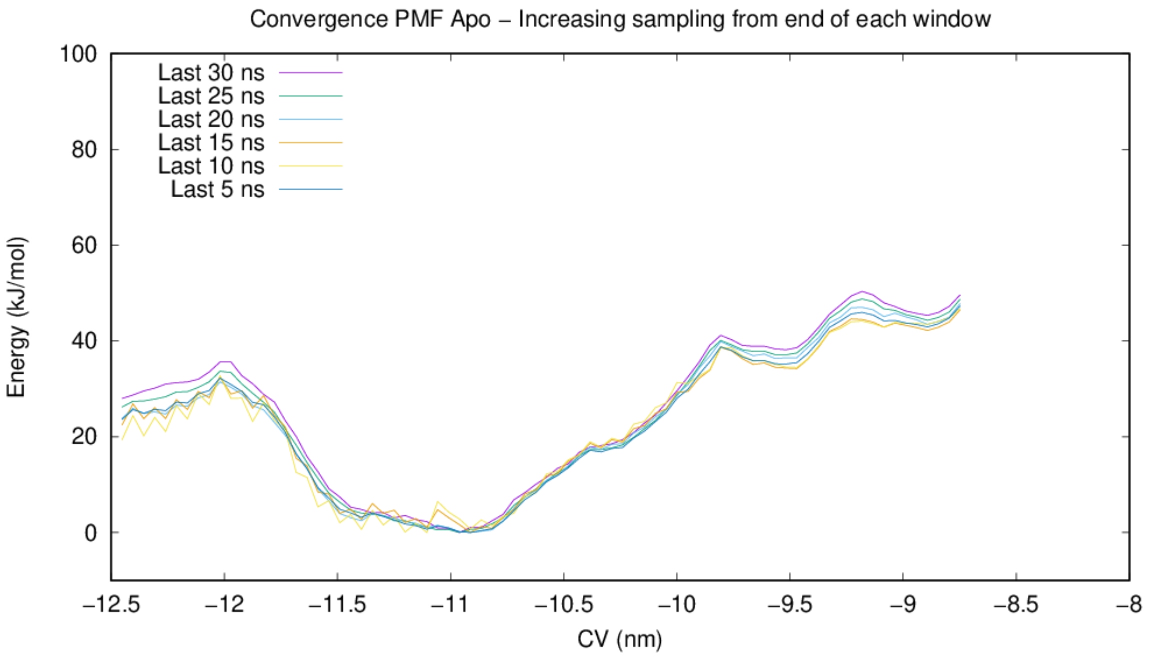

Figure S24- Evaluation of the Apo PMF convergence by increasingly adding 5 ns of sampling from the end of each umbrella window. The PMF using the last 5 ns of each window is represented in dark blue, using the last 10 ns of each window is represented in yellow, the last 15 ns of each window is represented in orange, the last20 ns of each window is represented in cyan, the last 25 ns of each window is represented in green and using the whole 30 ns of each window is represented in purple.

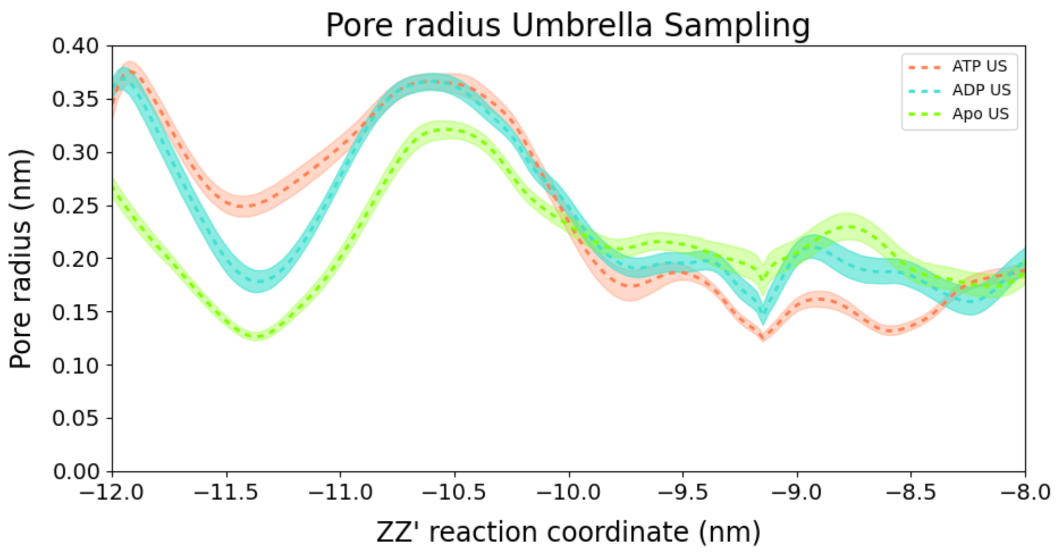

Figure S25-Average pore radius during the umbrella sampling simulations for the three simulated states: ATP, ADP and Apo. The error shading corresponds to the 95% confidence interval obtained with bootstrapping.

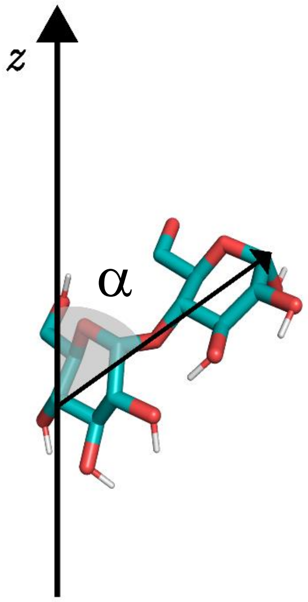

Figure S26- Angle used to determine maltose orientation. The angle is calculated between the z-axis and the vector that links the most distant carbon atoms in the maltose rings, covering the entirety of the maltose molecule.

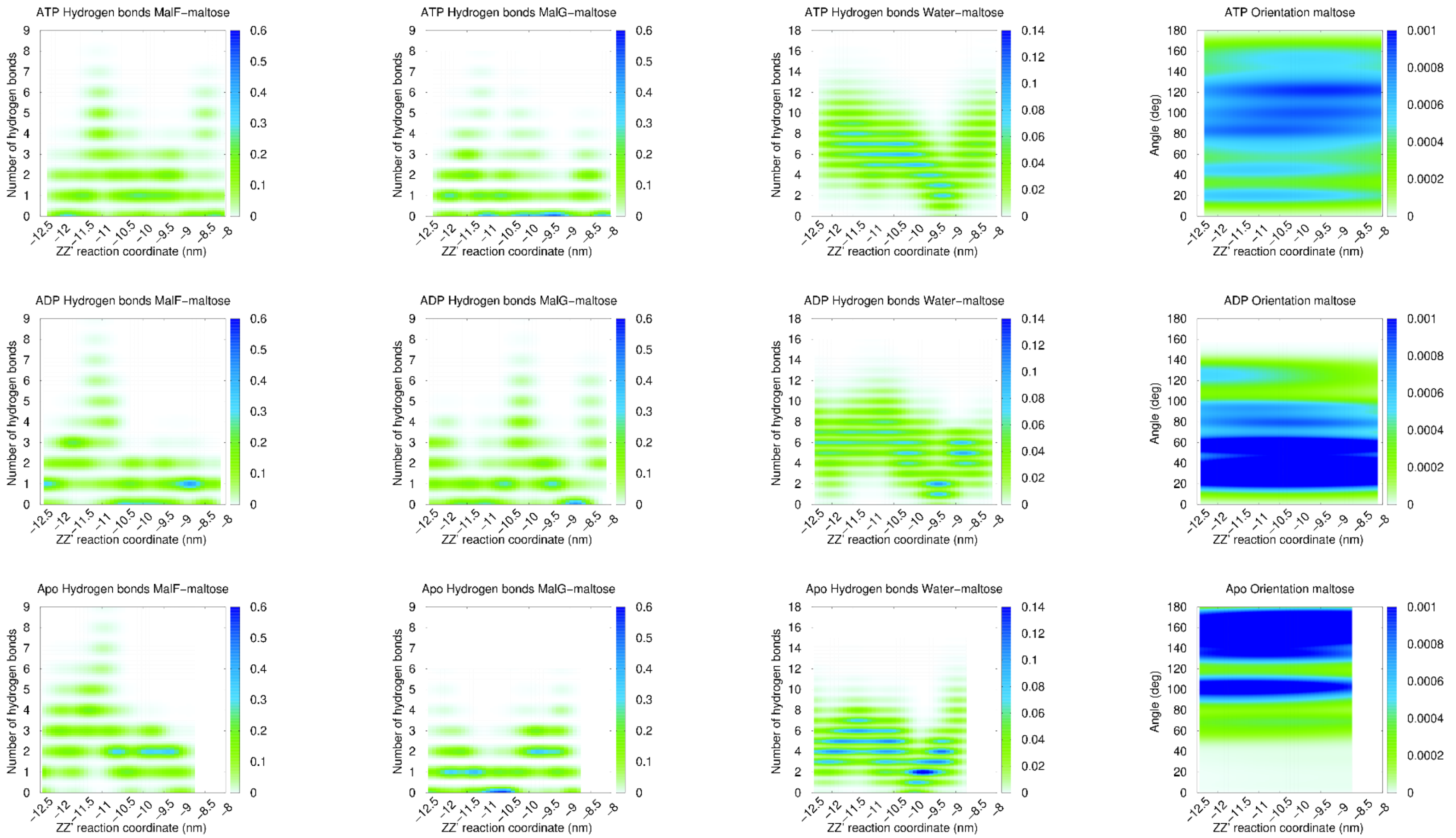

Figure S27- 2D histograms showing the variation of several properties related with maltose in function of the reaction coordinate. These are: the number of hydrogen bonds performed by maltose with the MalF, MalG and water molecules. The orientation angle of maltose in the pore is also represented. It is defined as the angle between the z-axis and the vector that links the most distant carbon atoms in the maltose rings. Angles between 80º and 120º reflect horizontal conformations, while angles between 140º and 170º or 20º to 60º reflect vertical conformations.
